# Structural characterization of three norfloxacin salts with N-heterocyclic mono- and di­carb­oxy­lic acids

**DOI:** 10.1107/S2056989026007589

**Published:** 2026-07-31

**Authors:** Anna Ben, Valeryia Hushcha, Lilianna Chęcińska

**Affiliations:** a University of Lodz Doctoral School of Exact and Natural Sciences Narutowicza 68 90-136 Łódź Poland; b University of Lodz Faculty of Chemistry Pomorska 163/165 90-236 Łódź Poland; Katholieke Universiteit Leuven, Belgium

**Keywords:** norfloxacin, crystal structure, mol­ecular salt, Hirshfeld surface analysis

## Abstract

Cocrystallization of norfloxacin (NFX) with three heterocyclic coformers, namely, pyridine-3,5-di­carb­oxy­lic acid, pyridazine-3-carb­oxy­lic acid and pyrimidine-5-carb­oxy­lic acid, yielded three mol­ecular salts: norfloxacinium [4-(3-carb­oxy-1-ethyl-6-fluoro-4-oxo-1,4-di­hydro­quinolin-7-yl)piperazin-1-ium] 5-carb­oxy­pyridine-3-carboxyl­ate trihydrate, 2C_16_H_19_FN_3_O_3_^+^·2C_7_H_4_NO_4_^−^·3H_2_O, (**1**), norfloxacinium pyridazine-3-carboxyl­ate, C_16_H_19_FN_3_O_3_^+^·C_5_H_3_N_2_O_2_^−^, (**2**), and norfloxacinium pyrimidine-5-carboxyl­ate monohydrate, C_16_H_19_FN_3_O_3_^+^·C_5_H_3_N_2_O_2_^−^·H_2_O, (**3**). In all structures, the quinolone skeleton of the norfloxacin cation is essentially planar, while the piperazine ring adopts a chair conformation. The crystal packing is governed primarily by N—H⋯O and N—H⋯N hydrogen bonds, which generate distinct mono-periodic and di-periodic motifs depending on the coformer. These assemblies are further linked by weak C—H⋯O and C—H⋯N inter­actions into tri-periodic supra­molecular networks. The crystal structures are additionally stabilized by aromatic π–π inter­actions, which differ in their stacking arrangements among the three salts. Hirshfeld surface analysis of the norfloxacin cations shows that H⋯H and O⋯H/H⋯O contacts dominate, whereas variations in the contributions from C⋯C, C⋯H/H⋯C and N⋯H/H⋯N contacts reflect differences in the supra­molecular packing.

## Abstract 2

Three new mol­ecular salts of norfloxacin with N-heterocyclic carboxyl­ates exhibit distinct hydrogen-bonding patterns and π-stacking arrangements, giving rise to different supra­molecular architectures.

## 1. Chemical context

Pharmaceutical multicomponent crystal forms, including co­crystals and mol­ecular salts, have become an important strategy in crystal engineering for improving the physicochemical properties of active pharmaceutical ingredients (APIs), such as solubility, dissolution behaviour, stability and bioavailability, without altering their pharmacological activity. The design of such crystalline materials relies on the rational manipulation of inter­molecular inter­actions and supra­molecular synthons to produce solids with predictable structural features and tailored properties (Desiraju, 1989 ▸, 1995 ▸; Bolla *et al.*, 2022 ▸).

Norfloxacin, 1-ethyl-6-fluoro-1,4-di­hydro-6-fluoro-4-oxo-7-(piperazin-1-yl)quinoline-3-carboxylic acid (NFX), a second-generation fluoro­quinolone discovered in the late 1970s, is a suitable candidate for multicomponent crystal design because of its poor aqueous solubility and low permeability. Consequently, it is classified as a Biopharmaceutics Classification System (BCS) class IV drug (Gunnam & Nangia, 2023 ▸). Norfloxacin is active against Gram-positive and Gram-negative bacteria (Zeng *et al.*, 2024 ▸) and is used in the treatment of bacterial infections of the prostate and the urinary and biliary tracts (Holmes *et al.*, 1985 ▸; Tabeta *et al.*, 1987 ▸; Lee & Wong, 1988 ▸).
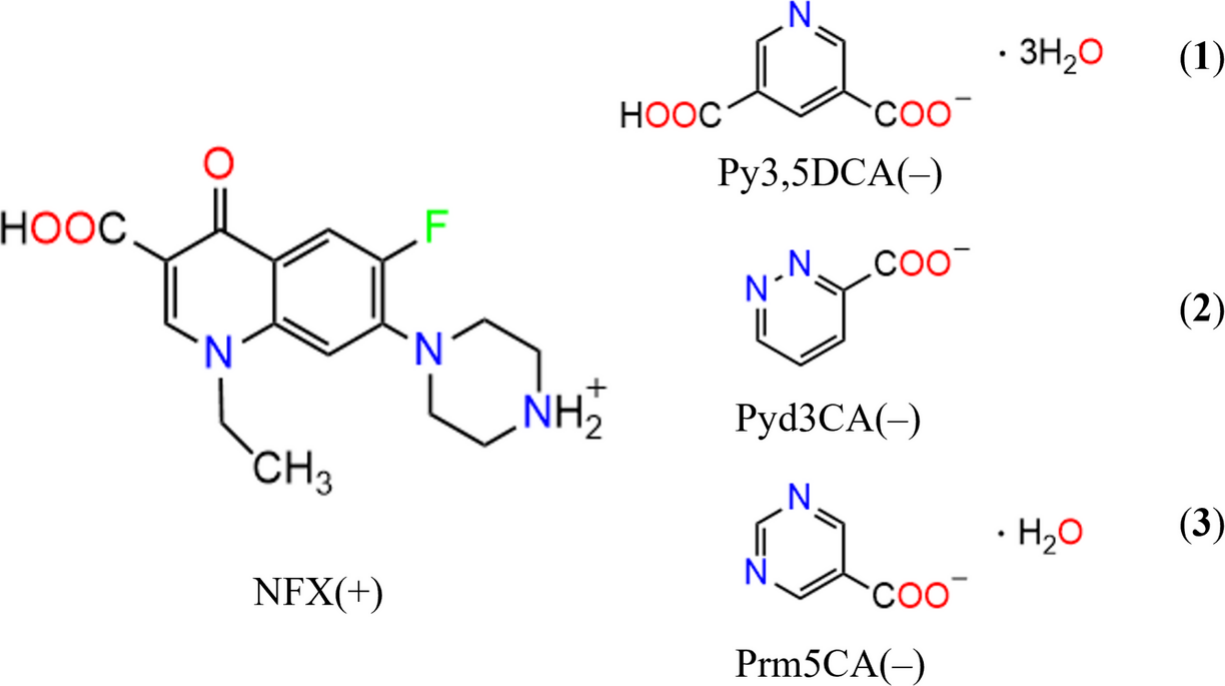


In this work, three new mol­ecular salts of norfloxacin with N-heterocyclic mono- and di­carb­oxy­lic acids have been prepared and structurally characterized. The selected coformers are pyridine-3,5-di­carb­oxy­lic acid (Py3,5DCA) pyridazine-3-carb­oxy­lic acid (Pyd3CA) and pyrimidine-5-carb­oxy­lic acid (Prm5CA). The crystal structures of three NFX multicomponent forms (**1**)–(**3**) are reported and discussed with emphasis on the inter­molecular inter­actions governing crystal packing.

## 2. Structural commentary

All three investigated multicomponent crystals of norfloxacin are mol­ecular salts. The formal positive charge is located on the nitro­gen atom (N3/N6) of the piperazine ring, while the negative charge is delocalized over the carboxyl­ate group of coformer monoanions.

**Figure 1:**
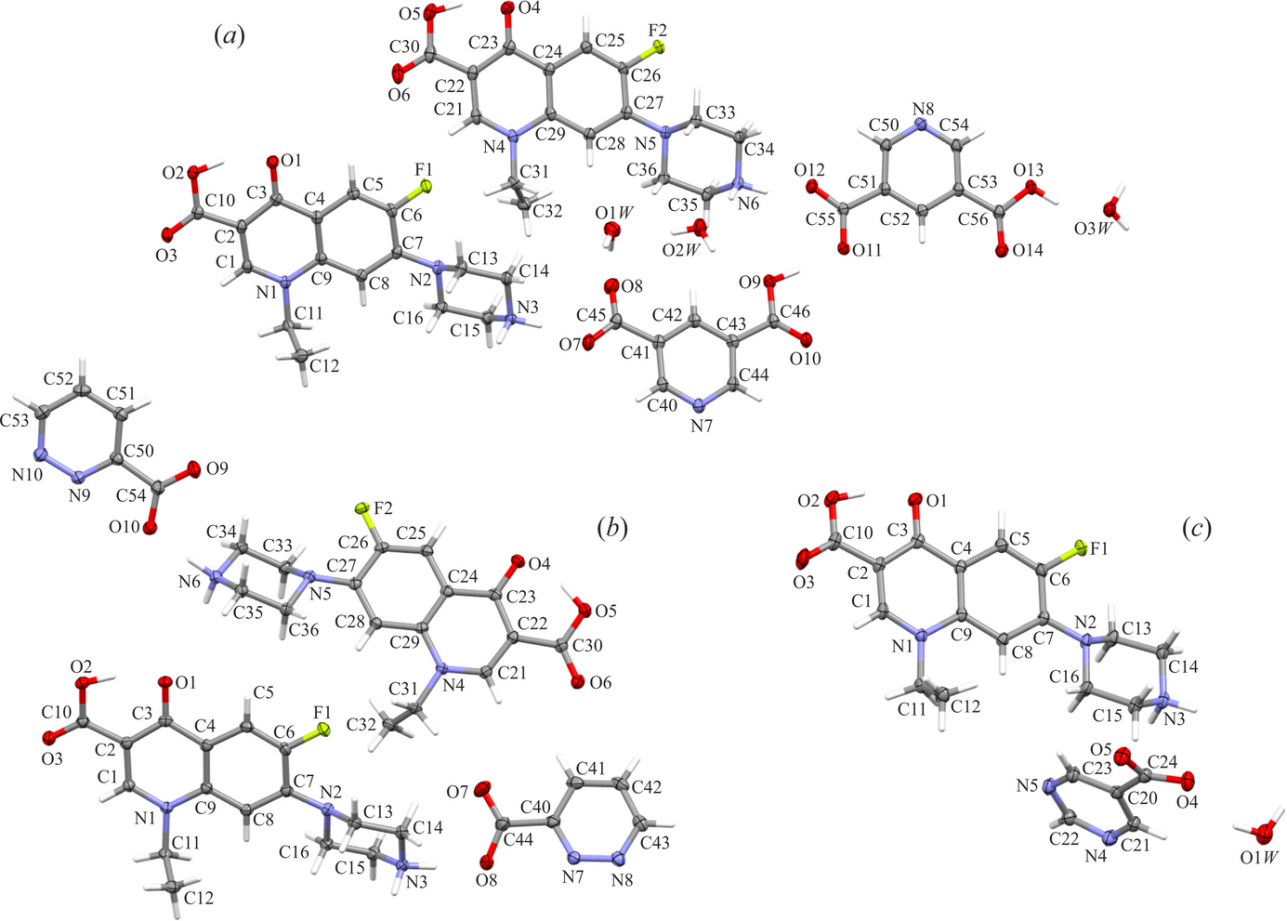
The mol­ecular structures of (*a*) 2NFX(+)·2Py3,5DCA(–)·3H_2_O (**1**), (*b*) NFX(+)·Pyd3CA(–) (**2**) and (*c*) NFX(+)·Prm5CA(–)·H_2_O (**3**) with the atom-numbering scheme. Displacement ellipsoids are drawn at the 50% probability level.

All three mol­ecular salts crystallize in the triclinic space group *P*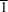. The asymmetric units (Fig. 1 ▸) of 2NFX(+)·2Py3,5DCA(–)·3H_2_O (**1**) and NFX(+)·Pyd3CA(–) (**2**) each comprise two norfloxacin cations and two coformer anions; in addition, the asymmetric unit of (**1**) contains three water mol­ecules. In contrast, the asymmetric unit of NFX(+)·Prm5CA(–)·H_2_O (**3**) consists of one norfloxacin cation, one pyrimidine-5-carboxyl­ate anion and one water mol­ecule.

The quinolone skeleton of the NFX mol­ecules remains essentially planar in all structures. The dihedral angle between the heterocyclic and benzene rings ranges from 1.67 (6) to 3.95 (6)°, confirming the planarity of the fused bicyclic system (Table 1 ▸). The torsion angle C1—N1—C11—C12, ranging from −99.62 (13) to −104.23 (11)°, indicates a nearly perpendicular orientation of the ethyl group with respect to the quinolone skeleton. The second independent NFX mol­ecule in (**2**) adopts the opposite orientation, with the torsion angle of 105.88 (13)°, showing the conformational flexibility of the ethyl group (Table 1 ▸).

**Figure 2:**
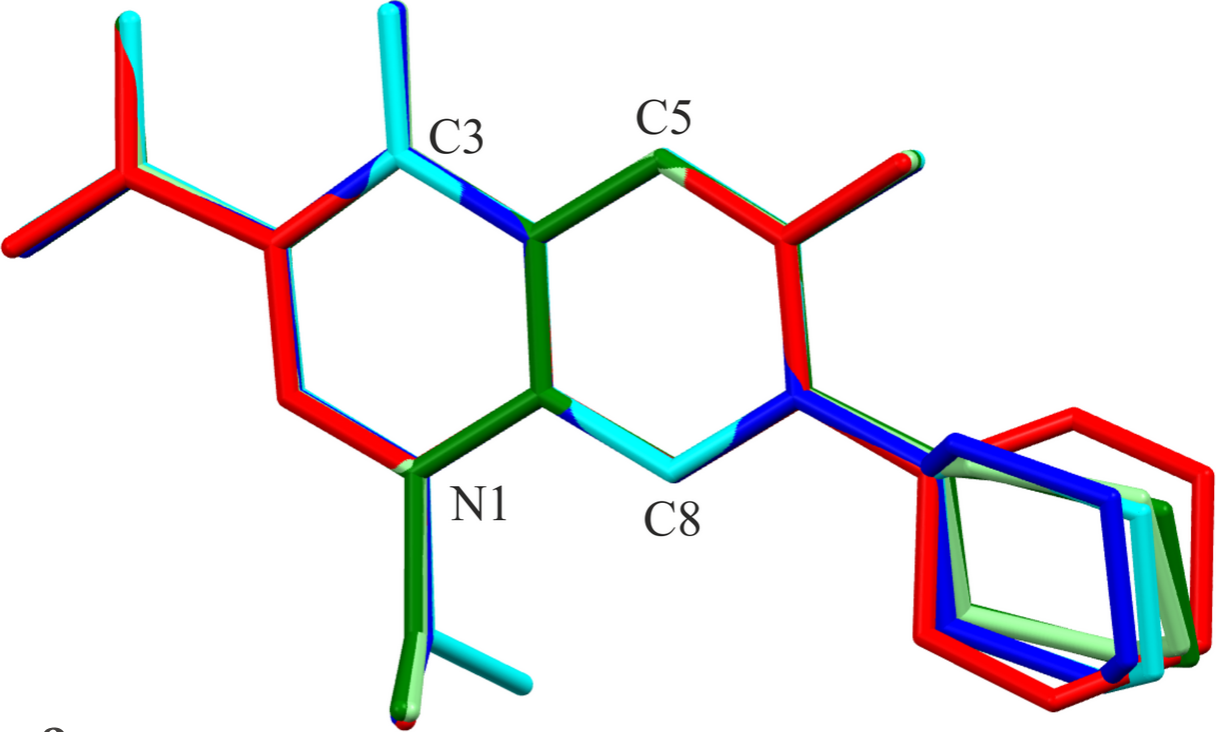
An overlay of five independent norfloxacin mol­ecules present in the title mol­ecular salts with the best fit for atoms N1, C3, C5 and C8. The color code is blue – mol­ecule 1 in (**1**); cyan – mol­ecule 2 in (**1**); green – mol­ecule 1 in (**2**), light green – mol­ecule 2 in (**2**) and red – mol­ecule in (**3**). The root mean-square (r.m.s.) deviation between two independent NFX mol­ecules is 0.484 Å for salt (**1**) and 0.142 Å for salt (**2**), calculated for the best fit of 23 non-hydrogen atoms (Spek, 2020 ▸).

The piperazine ring adopts a chair conformation, with the puckering amplitude (*Q*) lying within a narrow range of 0.5705 (12) – 0.5845 (14) Å. The θ values are close to 180° in structures (**1**) and (**2**); however, in structure (**3**) the opposite chair conformation is observed with θ close to 0° (Table 1 ▸). This difference is clearly illustrated by the superposition of the five independent NFX mol­ecules in Fig. 2 ▸.

In all investigated crystal structures, the quinolone moiety occupies an equatorial position of the piperazine ring. The angle between the C7—N2 bond and the normal to the Cremer & Pople mean plane of the piperazine ring varies from 79.72 (8) to 88.23 (7)° (Cremer & Pople, 1975 ▸). Consequently, the dihedral angle between these mol­ecular fragments ranges from 27.98 (5) to 44.70 (4)°.

## 3. Supra­molecular features

**Figure 3:**
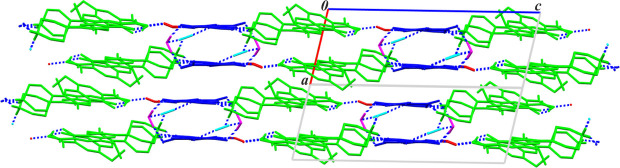
Crystal packing of 2NFX(+)·2Py3,5DCA(–)·3H_2_O (**1**) showing the di-periodic supra­molecular layers. Norfloxacin cations are shown in green, Py3,5DCA anions in blue and water mol­ecules in red, cyan and pink. (C)—H atoms not involved in hydrogen bonds have been omitted.

In the crystal structure of (**1**), Py3,5DCA(–) and water mol­ecules (O3*W*) form a hydrogen-bonded chain submotif with the sequence coformer-coformer-water. The remaining water mol­ecules (O1*W* and O2*W*) connect two neighbouring chains, generating centrosymmetric channels. The first NFX mol­ecule inter­acts with these chains through N—H⋯N_coformer_ and N—H⋯O_coformer_ hydrogen bonds (Table 2 ▸), whereas the second NFX mol­ecule forms N—H⋯O hydrogen bonds with both the coformer and water mol­ecules. Furthermore, the O3*W*—H2*W*3⋯O5 hydrogen bond links adjacent columns into a supra­molecular layer parallel to the (110) plane (Fig. 3 ▸).

**Figure 4:**
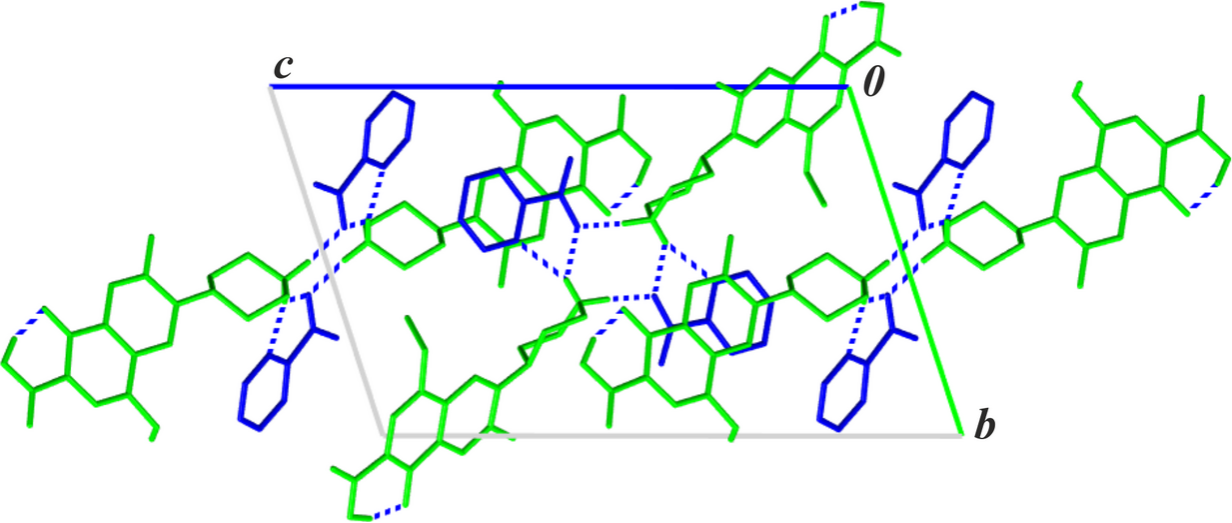
Crystal packing of NFX(+)·Pyd3CA(–) (**2**) showing the finite four-mol­ecule supra­molecular motifs. Norfloxacin cations are shown in green and Pyd3CA anions in blue. (C)—H atoms not involved in hydrogen bonds have been omitted.

In the crystal structure of (**2**), each NFX(+) mol­ecule inter­acts with Pyd3CA(–) coformer in the same manner. The terminal nitro­gen atom of the piperazine ring forms three hydrogen bonds, including one N—H⋯O_coformer_ hydrogen bond and one bifurcated donor hydrogen bond involving oxygen and nitro­gen atoms of the same coformer mol­ecule as acceptors (Table 3 ▸). These ionic pairs inter­act with each other forming finite supra­molecular motifs composed of three fused hydrogen-bonded rings: [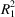
(5)
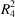
(8)
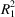
(5)] (Etter *et al.*, 1990 ▸; Bernstein *et al.*, 1995 ▸) (Fig. 4 ▸).

**Figure 5:**
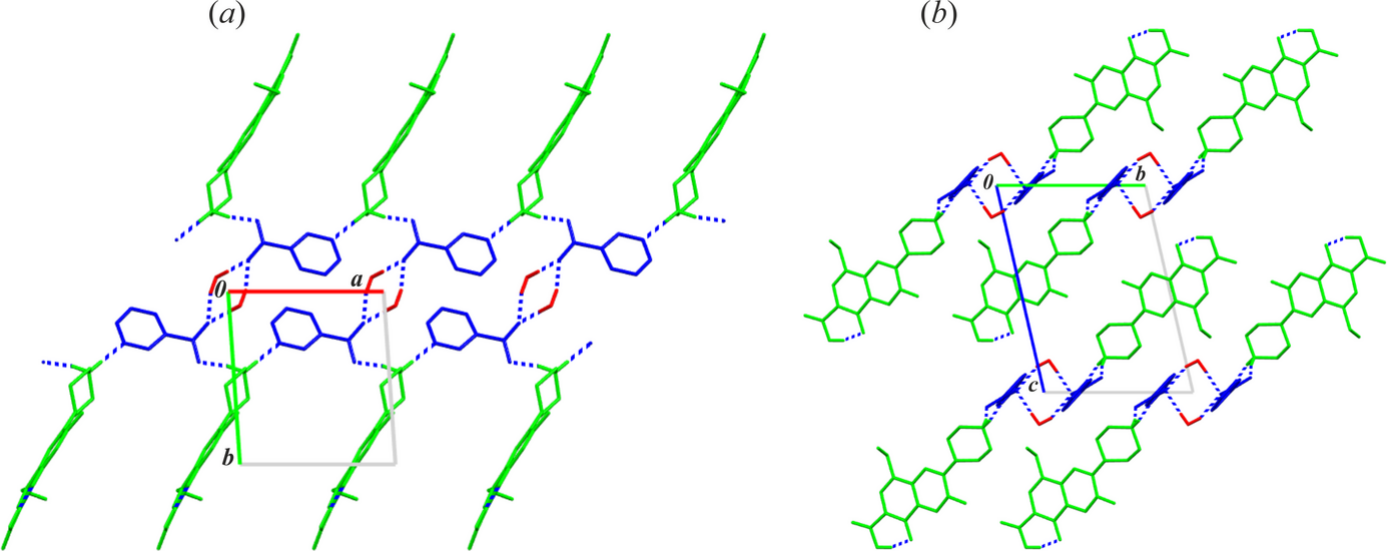
A part of the crystal structure of NFX(+)·Prm5CA(–)·H_2_O (**3**) showing (*a*) a hydrogen-bonded chain motif and (*b*) the mono-periodic ribbons. Norfloxacin cations are shown in green, Prm5CA anions in blue and water mol­ecules in red. (C)—H atoms not involved in hydrogen bonds have been omitted.

In the crystal structure of (**3**), the NFX(+) mol­ecules inter­act with Prm5CA(–) mol­ecules through N—H⋯N_coformer_ and N—H⋯O_coformer_ hydrogen bonds, generating chains (Table 4 ▸, Fig. 5 ▸*a*). Centrosymmetrically related water mol­ecules link two adjacent chains into a mono-periodic ribbon running along the [100] direction (Fig. 5 ▸*b*).

In all three crystal structures, (**1**)–(**3**), the hydrogen-bonded motifs described above are further connected by weak C—H⋯O and C—H⋯N inter­actions, resulting in tri-periodic supra­molecular networks. These supra­molecular architectures are further stabilized by aromatic π–π inter­actions (Table 5 ▸). However, each structure exhibits a different π-stacking arrangement. In (**1**), two types of mol­ecular stacks are observed, consisting exclusively of either NFX or coformer mol­ecules. In (**3**), only the NFX mol­ecules form mol­ecular stacks, in which the heterocyclic and benzene rings participate in π–π inter­actions. In contrast, in (**2**), aromatic π–π inter­actions occur only within isolated NFX–coformer pairs.

## 4. Hirshfeld surface analysis

**Figure 6:**
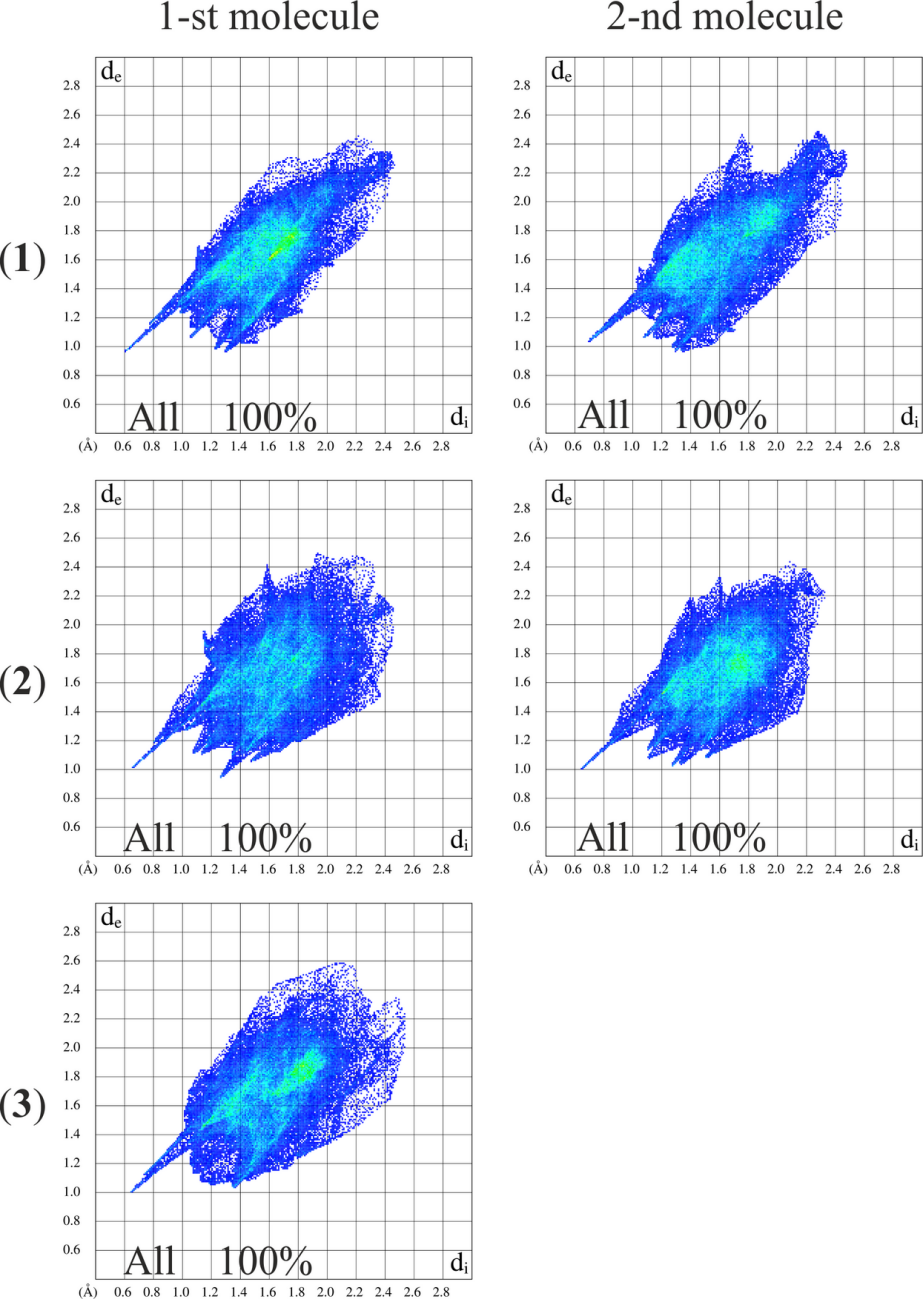
Two-dimensional fingerprint plots for the Hirshfeld surfaces of five independent norfloxacin mol­ecules in mol­ecular salts (**1**)–(**3**). The *d*_i_ and *d*_e_ values are the closest inter­nal and external distances (in Å) from given points on the Hirshfeld surface.

**Figure 7:**
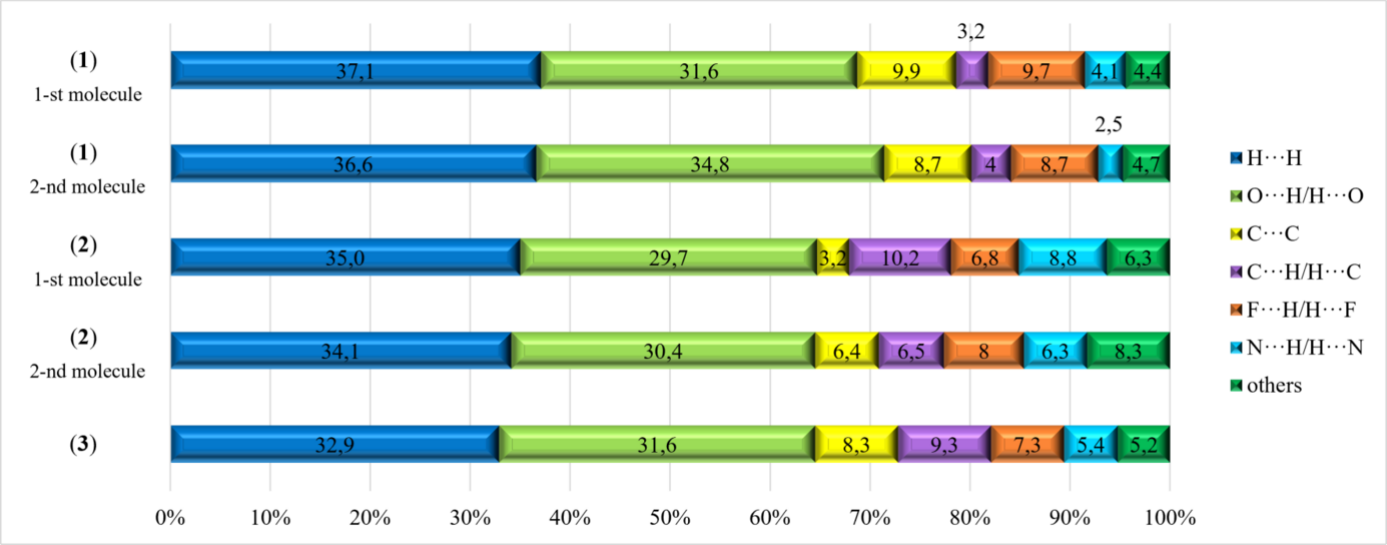
Diagram of the percentage contributions of different close contacts to the Hirshfeld surfaces of five independent norfloxacin mol­ecules in mol­ecular salts (**1**)–(**3**).

Hirshfeld surfaces and fingerprint plots (Spackman & McKinnon, 2002 ▸; Spackman & Jayatilaka, 2009 ▸) were generated with *CrystalExplorer* (Spackman *et al.*, 2021 ▸) to visualize and qu­antify inter­molecular inter­actions in three norfloxacin salts (Fig. 6 ▸). As shown in the fingerprint breakdown diagram (Fig. 7 ▸), the Hirshfeld surfaces of the NFX mol­ecules are dominated by H⋯H and O⋯H/H⋯O contacts. The contribution of F⋯H/H⋯F contacts is similar in all three salts (6.8–9.7%) indicating a comparable involvement of the fluorine atom in the crystal packing. The relatively high contribution of C⋯C contacts in salts (**1**) (8.7–9.9%) and (**3**) (8.3%) suggests more pronounced π-π- stacking inter­actions, whereas these contacts are considerably less significant for one of the mol­ecules in salt (**2**) (3.2%). An opposite trend is observed for N⋯H/H⋯N and C⋯H/H⋯C contacts, which reach their highest contributions to the Hirshfeld surface of the first independent NFX mol­ecule in salt (**2**), accounting for 8.8% and 10.2%, respectively. Each of the other contact types contributes less than 6.6% in multicomponent forms (**1**)–(**3**).

## 5. Database survey

A search of the Cambridge Structural Database (CSD version 6.01, February 2026; Groom *et al.*, 2016 ▸) revealed 16 crystal structures of multicomponent forms of norfloxacin containing benzoic acid derivatives as coformers (private communications have been omitted). Nine are mol­ecular salts with substituted benzoate anions (BOLVUX01; Li & Jiang, 2025 ▸; LOQLAG; LOQLOU; LOQLUA; Xu *et al.*, 2014 ▸; ORUYEG; Reddy *et al.*, 2011 ▸; WOXRUA01; WOXSAH01; Liang *et al.*, 2024 ▸; INOMOR; de Paula Costa *et al.*, 2026 ▸; LUVCOY01; Wang *et al.*, 2025 ▸), whereas the remaining seven contain carb­oxy­benzoate anions (HIGSAT; HIGSIB; HIGSUN; HIGTAU; UPAYOA01; Huang *et al.*, 2013 ▸; UPAYOA; Yan *et al.*, 2011 ▸; VOLMUI01; Sun *et al.*, 2024 ▸). Most of these crystal forms are hydrated, comprising nine monohydrates and two dihydrates.

A separate CSD search for multicomponent forms of norfloxacin with *N*-heteroaromatic coformers yielded only three entries: a cocrystal with pyridine-3-carb­oxy­lic acid (GADQEL; Ferreira *et al.*, 2020 ▸), a tetra­hydrate mol­ecular salt with a pyrazine-2-carboxyl­ate (UKIXOF; de Paula Costa *et al.*, 2025 ▸) and a chloro­form-solvated cocrystal with isonicotinamide, in which norfloxacin occurs in the zwitterionic form (VETVUM; Basavoju *et al.*, 2006 ▸).

## 6. Crystallization

Norfloxacin used in this study was purchased from TCI (Tokyo Chemical Industry Co., Ltd., Japan). Pyridine-3,5-di­carb­oxy­lic acid, pyridazine-3-carb­oxy­lic acid and pyrimidine-5-carb­oxy­lic acid were obtained from Fluoro­chem (Hadfield, United Kingdom). Ethanol (analytical grade; Chempur, Piekary Śląskie, Poland) was used as received.

Equimolar amounts of norfloxacin and the appropriate coformer were ground together and dissolved in ethanol. The resulting mixtures were heated under stirring until complete dissolution, filtered and left to evaporate slowly at ambient temperature. Single crystals suitable for X-ray diffraction were obtained from the filtrates.

## 7. Refinement

Crystal data, data collection and structure refinement details are summarized in Table 6 ▸. The H atoms bonded to C atoms were included in the refinement using riding models with constrained distances set to 0.95 Å (aromatic), 0.98 Å (meth­yl), 0.99 Å (methyl­ene), and with *U*_iso_(H) = *kU*_eq_(C), where *k*=1.5 for the methyl group and 1.2 for all other H atoms bonded to C atoms. The hydrogen atoms bonded to heteroatoms (N and O) involved in hydrogen bonds were located in difference Fourier maps and refined isotropically.

## Supplementary Material

Crystal structure: contains datablock(s) 1, 2, 3, global. DOI: 10.1107/S2056989026007589/vm2333sup1.cif

Structure factors: contains datablock(s) 1. DOI: 10.1107/S2056989026007589/vm23331sup5.hkl

Structure factors: contains datablock(s) 2. DOI: 10.1107/S2056989026007589/vm23332sup6.hkl

Structure factors: contains datablock(s) 3. DOI: 10.1107/S2056989026007589/vm23333sup7.hkl

Supporting information file. DOI: 10.1107/S2056989026007589/vm23331sup5.cml

Supporting information file. DOI: 10.1107/S2056989026007589/vm23332sup6.cml

Supporting information file. DOI: 10.1107/S2056989026007589/vm23333sup7.cml

CCDC references: 2575687, 2575686, 2575685

Additional supporting information:  crystallographic information; 3D view; checkCIF report

## Figures and Tables

**Table 1:** Geometric parameters describing the mol­ecular conformation of norfloxacin in mol­ecular salts (**1**)–(**3**) C1—N1—C11—C12 or C21—N4—C31—C32 torsion angle (°) defining the orientation of the ethyl group to heterocyclic ring of the norfloxacin mol­ecule; Cremer & Pople puckering parameters defining the chair conformation of the piperazine ring: *Q* (Å) – the puckering amplitude; the angular parameters θ (°) and φ (°); Analysis of ring substituent in the piperazine ring: the angle (°) between the C7—N2 or C27—N5 bond and the normal to Cremer & Pople plane; dihedral angle (°) between the best planes of heterocyclic and benzene rings, forming the ten-membered quinolone system; dihedral angle (°) between the best planes of the ten-membered quinolone system and the piperazine ring.

Structure	Torsion angle	*Q*	θ	φ	Angle C7—N2 bond/CP plane	Dihedral angle heterocyclic/benzene	Dihedral angle quinolone/piperazine
(**1**)	–99.62 (13)	0.5782 (14)	180.00 (13)	159 (21)	79.72 (8)	1.67 (6)	40.55 (5)
	105.88 (13)	0.5727 (13)	178.13 (14)	96 (4)	83.11 (8)	2.97 (6)	37.45 (5)
(**2**)	–104.20 (11)	0.5705 (12)	175.26 (11)	167.5 (15)	85.91 (7)	3.64 (5)	43.77 (5)
	–104.23 (11)	0.5730 (12)	171.82 (11)	183.4 (9)	88.23 (7)	2.55 (5)	44.70 (4)
(**3**)	–100.30 (13)	0.5845 (14)	3.88 (14)	47 (2)	86.86 (8)	3.95 (6)	27.98 (5)

**Table 2:** Hydrogen-bond geometry (Å, °) for (**1**)

*D*—H⋯*A*	*D*—H	H⋯*A*	*D*⋯*A*	*D*—H⋯*A*
O2—H2⋯O1	0.97 (2)	1.64 (2)	2.5554 (13)	156 (2)
O5—H5*A*⋯O4	0.98 (3)	1.54 (3)	2.4830 (14)	158 (2)
O9—H9⋯O11	0.95 (2)	1.69 (3)	2.6396 (13)	176 (2)
O13—H13⋯O3*W*	0.93 (2)	1.75 (2)	2.6342 (14)	157 (2)
N3—H3*B*⋯O7	0.94 (2)	1.66 (2)	2.5925 (14)	170 (2)
N3—H3*A*⋯N8^i^	0.945 (19)	2.009 (19)	2.8825 (16)	153 (2)
N6—H6*A*⋯O2*W*	0.887 (18)	1.908 (19)	2.7646 (15)	162 (2)
N6—H6*B*⋯O12	0.94 (2)	1.81 (2)	2.6856 (14)	154 (2)
C5—H5⋯F1^ii^	0.95	2.32	3.0193 (15)	130
C11—H11*B*⋯O6^iii^	0.99	2.35	3.2564 (16)	152
C12—H12*B*⋯F2^i^	0.98	2.36	3.2576 (15)	151
C15—H15*A*⋯O11^iv^	0.99	2.38	3.3290 (16)	161
C33—H33*A*⋯O2^ii^	0.99	2.36	3.2282 (15)	145
O1*W*—H1*W*1⋯O8	0.95 (2)	1.76 (2)	2.7047 (2)	174 (2)
O1*W*—H2*W*1⋯O14^v^	0.90 (2)	1.98 (2)	2.8834 (2)	173 (2)
O2*W*—H1*W*2⋯O1*W*	0.84 (2)	1.97 (2)	2.7974 (16)	167 (2)
O2*W*—H2*W*2⋯O11^v^	0.88 (2)	1.95 (2)	2.8171 (14)	166 (2)
O3*W*—H1*W*3⋯N7^vi^	0.87 (2)	1.96 (2)	2.7937 (15)	161 (2)
O3*W*—H2*W*3⋯O5^vii^	0.83 (3)	2.41 (2)	3.0846 (14)	138 (2)

Symmetry codes: (i) 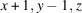; (ii) 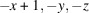; (iii) 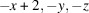; (iv) 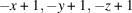; (v) 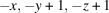; (vi) 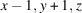; (vii) 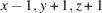.

**Table 3:** Hydrogen-bond geometry (Å, °) for (**2**)

*D*—H⋯*A*	*D*—H	H⋯*A*	*D*⋯*A*	*D*—H⋯*A*
O2—H2⋯O1	0.946 (18)	1.610 (18)	2.5174 (10)	159 (2)
O5—H5*A*⋯O4	0.970 (19)	1.592 (18)	2.5208 (11)	159 (2)
N3—H3*B*⋯O8	0.987 (17)	1.685 (18)	2.6682 (12)	174 (2)
N6—H6*B*⋯O10	0.993 (17)	1.668 (17)	2.6591 (12)	176 (2)
N3—H3*A*⋯O8^i^	0.921 (16)	2.254 (15)	2.8557 (13)	122 (1)
N3—H3*A*⋯N7^i^	0.921 (16)	2.072 (15)	2.9552 (13)	160 (1)
N6—H6*A*⋯O10^ii^	0.882 (15)	2.112 (14)	2.7721 (12)	131 (1)
N6—H6*A*⋯N9^ii^	0.882 (15)	2.194 (15)	3.0060 (13)	153 (1)
C11—H11*B*⋯O9^iii^	0.99	2.46	3.2870 (13)	141
C11—H11*A*⋯F2^iii^	0.99	2.37	3.2083 (12)	142
C16—H16*B*⋯N8^i^	0.99	2.50	3.4430 (14)	158
C36—H36*B*⋯N10^ii^	0.99	2.49	3.4388 (14)	160
C41—H41⋯O6	0.95	2.45	3.1048 (13)	126
C51—H51⋯O3^iv^	0.95	2.31	3.1186 (13)	143

Symmetry codes: (i) 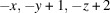; (ii) 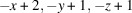; (iii) 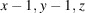; (iv) 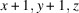.

**Table 4:** Hydrogen-bond geometry (Å, °) for (**3**)

*D*—H⋯*A*	*D*—H	H⋯*A*	*D*⋯*A*	*D*—H⋯*A*
O2—H2⋯O1	0.96 (2)	1.60 (3)	2.5234 (13)	160 (2)
N3—H3*A*⋯O5	0.98 (2)	1.67 (2)	2.6378 (15)	170 (2)
N3—H3*B*⋯N5^i^	0.94 (2)	1.93 (2)	2.8654 (16)	172 (2)
C1—H1⋯O3^ii^	0.95	2.48	3.2305 (16)	136
C11—H11*A*⋯N4^iii^	0.99	2.58	3.3630 (17)	136
C13—H13*A*⋯F1	0.99	2.20	2.8752 (15)	124
O1*W*—H1*W*⋯O4	0.91 (2)	2.04 (2)	2.8863 (16)	155 (2)
O1*W*—H2*W*⋯O4^iv^	0.94 (3)	2.01 (3)	2.9487 (17)	174 (2)

Symmetry codes: (i) 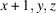; (ii) 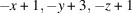; (iii) 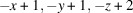; (iv) 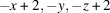.

**Table 5:** Geometric parameters (Å, °) of aromatic π–π stacking inter­actions for norfloxacin salts (**1**)–(**3**) *Cg*1/*Cg*2 are the centroids of the heterocyclic ring in NFX; *Cg*3/*Cg*4 are the centroids of the benzene ring in NFX; *Cg*5/*Cg*6 are the centroids of the aromatic ring in coformers (**1**)–(**3**). *CgI*⋯*CgJ* – distance between ring centroids; α – dihedral angle between planes *I* and *J*; *CgI*_perp_ and *CgJ*_perp_ (inter­planar spacing) – perpendicular distance of *CgI* on ring *J* and *CgJ* on ring *I*, respectively; slippage – distance between *CgI* and perpendicular projection of *CgJ* on ring *I*.

Structure	Contact	*CgI*⋯*CgJ*	α	*CgI*_perp_	*CgJ*_perp_	Slippage
(**1**)	*Cg*1⋯*Cg*1^iii^	3.4778 (7)	0.00 (6)	3.2329 (5)	3.2329 (5)	1.282
	*Cg*1⋯*Cg*3^iii^	3.6013 (7)	1.67 (6)	3.2152 (5)	3.2500 (5)	1.552
	*Cg*2⋯*Cg*2^viii^	3.8634 (7)	0.03 (6)	3.5678 (5)	3.5679 (5)	1.482
	*Cg*6⋯*Cg*5^v^	3.7431 (7)	6.68 (6)	3.3319 (5)	3.4232 (5)	1.514
	*Cg*6⋯*Cg*6^ix^	3.7718 (7)	0.00 (6)	3.5725 (5)	3.5724 (5)	1.210
(**2**)	*Cg*1⋯*Cg*6^ii^	3.6213 (6)	7.40 (5)	3.3032 (4)	3.4289 (5)	1.164
	*Cg*3⋯*Cg*6^ii^	3.6826 (7)	9.90 (5)	3.3350 (4)	3.4546 (5)	1.276
	*Cg*4⋯*Cg*5^v^	3.7303 (7)	8.10 (5)	3.0801 (4)	3.3419 (5)	1.657
	*Cg*5⋯*Cg*2^v^	3.5006 (6)	8.98 (5)	3.3215 (5)	3.4353 (4)	0.673
(**3**)	*Cg*1⋯*Cg*1^v^	3.5480 (7)	0.00 (6)	3.4031 (5)	3.4031 (5)	1.003
	*Cg*1⋯*Cg*3^v^	3.8093 (7)	3.95 (6)	3.3998 (5)	3.4876 (5)	1.532
	*Cg*3⋯*Cg*3^vi^	3.7265 (7)	0.00 (6)	3.6818 (5)	3.6819 (5)	0.575

Symmetry codes: (**1**) (iii) −*x* + 2, −*y*, −*z*; (viii) −*x* + 1, −*y* + 1, −*z*; (v) −*x*, −*y* + 1, −*z* + 1; (ix) −*x*, −*y* + 2, −*z* + 1; (**2**) (ii) −*x* + 2, −*y* + 1, −*z* + 1; (v) −*x* + 1, −*y* + 2, −*z* + 2; (**3**) (v) −*x* + 1, −*y* + 2, −*z* + 1; (vi) −*x* + 2, −*y* + 2, −*z* + 1.

**Table 6:** Experimental details

	(**1**)	(**2**)	(**3**)
Crystal data			
Chemical formula	2C_16_H_19_FN_3_O_3_^+^·2C_7_H_4_NO_4_^−^·3H_2_O	C_16_H_19_FN_3_O_3_^+^·C_5_H_3_N_2_O_2_^−^	C_16_H_19_FN_3_O_3_^+^·C_5_H_3_N_2_O_2_^−^·H_2_O
*M*_r_	1026.95	443.43	461.45
Crystal system, space group	Triclinic, *P* 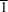	Triclinic, *P* 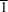	Triclinic, *P* 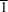
Temperature (K)	100	100	100
*a*, *b*, *c* (Å)	9.7162 (2), 12.5261 (2), 19.3460 (4)	9.0971 (1), 12.9566 (1), 19.2412 (2)	8.3657 (1), 9.5115 (1), 13.6068 (2)
α, β, γ (°)	96.772 (2), 99.257 (1), 99.128 (1)	104.666 (1), 95.813 (1), 110.485 (1)	76.490 (1), 82.771 (1), 84.520 (1)
*V* (Å^3^)	2269.44 (8)	2009.65 (4)	1041.92 (2)
*Z*	2	4	2
Radiation type	Cu *K*α	Cu *K*α	Cu *K*α
μ (mm^−1^)	1.03	0.95	0.98
Crystal size (mm)	0.18 × 0.12 × 0.04	0.25 × 0.19 × 0.05	0.22 × 0.17 × 0.04

Data collection			
Diffractometer	Rigaku, XtaLAB Synergy, Dualflex, HyPix	Rigaku, XtaLAB Synergy, Dualflex, HyPix	Rigaku, XtaLAB Synergy, Dualflex, HyPix
Absorption correction	Gaussian (*CrysAlis PRO*; Rigaku OD, 2025 ▸)	Gaussian (*CrysAlis PRO*; Rigaku OD, 2025 ▸)	Gaussian (*CrysAlis PRO*; Rigaku OD, 2025 ▸)
*T*_min_, *T*_max_	0.586, 1.000	0.626, 1.000	0.660, 1.000
No. of measured, independent and observed [*I* > 2σ(*I*)] reflections	28003, 9032, 8052	45406, 8113, 7634	31025, 4184, 3922
*R*_int_	0.031	0.019	0.023
(sin θ/λ)_max_ (Å^−1^)	0.633	0.633	0.632

Refinement			
*R*[*F*^2^ > 2σ(*F*^2^)], *wR*(*F*^2^), *S*	0.036, 0.104, 1.04	0.031, 0.084, 1.05	0.037, 0.097, 1.07
No. of reflections	9032	8113	4184
No. of parameters	716	603	319
No. of restraints	2	0	0
H-atom treatment	H atoms treated by a mixture of independent and constrained refinement	H atoms treated by a mixture of independent and constrained refinement	H atoms treated by a mixture of independent and constrained refinement
Δρ_max_, Δρ_min_ (e Å^−3^)	0.71, −0.27	0.25, −0.21	0.31, −0.22

Computer programs: *CrysAlis PRO* (Rigaku OD, 2025 ▸), *SHELXT* (Sheldrick, 2015*a* ▸), *SHELXL2025/1* (Sheldrick, 2015*b* ▸), *Mercury* (Macrae *et al.*, 2020 ▸) and *publCIF* (Westrip, 2010 ▸).

## supplementary crystallographic information

### 4-(3-Carboxy-1-ethyl-6-fluoro-4-oxo-1,4-dihydroquinolin-7-yl)piperazin-1-ium 5-carboxy-pyridine-3-carboxylate trihydrate (1) Crystal data

**Table:**

2C_16_H_19_FN_3_O_3_^+^·2C_7_H_4_NO_4_^−^·3(H_2_O)	*Z* = 2
*M**_r_* = 1026.95	*F*(000) = 1076
Triclinic, *P*1	*D*_x_ = 1.503 Mg m^−^^3^
*a* = 9.7162 (2) Å	Cu *K*α radiation, λ = 1.54184 Å
*b* = 12.5261 (2) Å	Cell parameters from 16961 reflections
*c* = 19.3460 (4) Å	θ = 3.6–76.5°
α = 96.772 (2)°	µ = 1.03 mm^−^^1^
β = 99.257 (1)°	*T* = 100 K
γ = 99.128 (1)°	Plate, colourless
*V* = 2269.44 (8) Å^3^	0.18 × 0.12 × 0.04 mm

### 4-(3-Carboxy-1-ethyl-6-fluoro-4-oxo-1,4-dihydroquinolin-7-yl)piperazin-1-ium 5-carboxy-pyridine-3-carboxylate trihydrate (1) Data collection

**Table:**

Rigaku, XtaLAB Synergy, Dualflex, HyPix diffractometer	9032 independent reflections
Radiation source: micro-focus sealed X-ray tube, PhotonJet (Cu) X-ray Source	8052 reflections with *I* > 2σ(*I*)
Mirror monochromator	*R*_int_ = 0.031
Detector resolution: 10.0000 pixels mm^-1^	θ_max_ = 77.5°, θ_min_ = 3.6°
ω scans	*h* = −11→12
Absorption correction: gaussian (CrysAlisPro; Rigaku OD, 2025)	*k* = −14→15
*T*_min_ = 0.586, *T*_max_ = 1.000	*l* = −24→17
28003 measured reflections	

### 4-(3-Carboxy-1-ethyl-6-fluoro-4-oxo-1,4-dihydroquinolin-7-yl)piperazin-1-ium 5-carboxy-pyridine-3-carboxylate trihydrate (1) Refinement

**Table:**

Refinement on *F*^2^	Primary atom site location: structure-invariant direct methods
Least-squares matrix: full	Hydrogen site location: mixed
*R*[*F*^2^ > 2σ(*F*^2^)] = 0.036	H atoms treated by a mixture of independent and constrained refinement
*wR*(*F*^2^) = 0.104	*w* = 1/[σ^2^(*F*_o_^2^) + (0.0565*P*)^2^ + 0.7761*P*] where *P* = (*F*_o_^2^ + 2*F*_c_^2^)/3
*S* = 1.04	(Δ/σ)_max_ < 0.001
9032 reflections	Δρ_max_ = 0.71 e Å^−^^3^
716 parameters	Δρ_min_ = −0.27 e Å^−^^3^
2 restraints	

### 4-(3-Carboxy-1-ethyl-6-fluoro-4-oxo-1,4-dihydroquinolin-7-yl)piperazin-1-ium 5-carboxy-pyridine-3-carboxylate trihydrate (1) Special details

**Table:**

Geometry. All esds (except the esd in the dihedral angle between two l.s. planes) are estimated using the full covariance matrix. The cell esds are taken into account individually in the estimation of esds in distances, angles and torsion angles; correlations between esds in cell parameters are only used when they are defined by crystal symmetry. An approximate (isotropic) treatment of cell esds is used for estimating esds involving l.s. planes.

### 4-(3-Carboxy-1-ethyl-6-fluoro-4-oxo-1,4-dihydroquinolin-7-yl)piperazin-1-ium 5-carboxy-pyridine-3-carboxylate trihydrate (1) Fractional atomic coordinates and isotropic or equivalent isotropic displacement parameters (Å^2^)

**Table:**

	*x*	*y*	*z*	*U*_iso_*/*U*_eq_	
F1	0.57815 (8)	0.05966 (6)	0.06233 (4)	0.02211 (17)	
O1	0.80626 (9)	−0.15482 (7)	−0.10697 (5)	0.01858 (18)	
O2	0.98509 (10)	−0.25471 (7)	−0.15846 (5)	0.01963 (19)	
O3	1.17176 (10)	−0.28635 (8)	−0.08633 (5)	0.0232 (2)	
N1	1.06397 (11)	−0.11602 (8)	0.08782 (5)	0.0164 (2)	
N2	0.72793 (11)	0.07193 (8)	0.19531 (5)	0.0169 (2)	
N3	0.63609 (12)	0.10385 (9)	0.32831 (6)	0.0193 (2)	
C1	1.09586 (13)	−0.17183 (10)	0.03083 (7)	0.0169 (2)	
H1	1.180717	−0.201089	0.036536	0.020*	
C2	1.01246 (13)	−0.18906 (9)	−0.03545 (6)	0.0166 (2)	
C3	0.88432 (13)	−0.14612 (9)	−0.04702 (6)	0.0159 (2)	
C4	0.84887 (13)	−0.08926 (9)	0.01590 (6)	0.0157 (2)	
C5	0.72535 (13)	−0.04342 (10)	0.01017 (7)	0.0169 (2)	
H5	0.665012	−0.049800	−0.034490	0.020*	
C6	0.69282 (13)	0.01007 (10)	0.06889 (7)	0.0173 (2)	
C7	0.77587 (13)	0.02058 (10)	0.13735 (6)	0.0163 (2)	
C8	0.90041 (13)	−0.02048 (10)	0.14252 (6)	0.0166 (2)	
H8	0.961326	−0.011885	0.187208	0.020*	
C9	0.93817 (13)	−0.07492 (9)	0.08235 (6)	0.0159 (2)	
C10	1.06412 (13)	−0.24769 (10)	−0.09447 (7)	0.0175 (2)	
C11	1.16011 (14)	−0.10326 (11)	0.15723 (7)	0.0197 (3)	
H11A	1.169190	−0.028044	0.182104	0.024*	
H11B	1.255448	−0.114408	0.149545	0.024*	
C12	1.10463 (16)	−0.18459 (11)	0.20290 (7)	0.0250 (3)	
H12A	1.165672	−0.170393	0.249749	0.038*	
H12B	1.104607	−0.258996	0.180550	0.038*	
H12C	1.007747	−0.176947	0.207913	0.038*	
C13	0.59338 (14)	0.00870 (10)	0.20668 (7)	0.0210 (3)	
H13A	0.610827	−0.060841	0.222896	0.025*	
H13B	0.524681	−0.009059	0.161439	0.025*	
C14	0.53187 (14)	0.07334 (11)	0.26124 (7)	0.0202 (3)	
H14A	0.507355	0.140163	0.243492	0.024*	
H14B	0.444055	0.028938	0.269709	0.024*	
C15	0.77156 (14)	0.16682 (10)	0.31686 (7)	0.0204 (3)	
H15A	0.840660	0.183929	0.361977	0.025*	
H15B	0.754883	0.236656	0.300860	0.025*	
C16	0.83151 (14)	0.10108 (10)	0.26164 (7)	0.0188 (3)	
H16A	0.919851	0.144628	0.253081	0.023*	
H16B	0.854825	0.033789	0.279166	0.023*	
F2	0.07596 (8)	0.60893 (6)	0.07893 (4)	0.02097 (16)	
O4	0.28416 (11)	0.41374 (8)	−0.10935 (5)	0.0275 (2)	
O5	0.43829 (12)	0.30850 (9)	−0.16961 (5)	0.0314 (2)	
O6	0.59023 (11)	0.22416 (9)	−0.10910 (6)	0.0312 (2)	
N4	0.46059 (11)	0.33020 (8)	0.07957 (6)	0.0174 (2)	
N5	0.17230 (11)	0.54148 (8)	0.20568 (5)	0.0163 (2)	
N6	0.06828 (11)	0.56020 (9)	0.33654 (6)	0.0177 (2)	
C21	0.50266 (13)	0.29701 (10)	0.01929 (7)	0.0193 (3)	
H21	0.574332	0.253360	0.021219	0.023*	
C22	0.44701 (14)	0.32307 (10)	−0.04555 (7)	0.0194 (3)	
C23	0.34107 (14)	0.38969 (10)	−0.05079 (7)	0.0195 (3)	
C24	0.30098 (13)	0.42960 (10)	0.01522 (7)	0.0173 (2)	
C25	0.20360 (14)	0.50162 (10)	0.01617 (7)	0.0183 (2)	
H25	0.165573	0.526262	−0.026217	0.022*	
C26	0.16448 (13)	0.53560 (10)	0.07835 (7)	0.0169 (2)	
C27	0.21476 (13)	0.50077 (10)	0.14325 (6)	0.0160 (2)	
C28	0.31211 (13)	0.43118 (10)	0.14203 (7)	0.0171 (2)	
H28	0.348283	0.405932	0.184508	0.020*	
C29	0.35846 (13)	0.39715 (10)	0.07921 (7)	0.0165 (2)	
C30	0.49918 (14)	0.28049 (11)	−0.10966 (7)	0.0234 (3)	
C31	0.52326 (14)	0.29572 (10)	0.14671 (7)	0.0201 (3)	
H31A	0.572282	0.234058	0.135600	0.024*	
H31B	0.446194	0.269378	0.171749	0.024*	
C32	0.62818 (15)	0.38788 (11)	0.19540 (7)	0.0238 (3)	
H32A	0.656093	0.364436	0.241380	0.036*	
H32B	0.583635	0.452369	0.201985	0.036*	
H32C	0.712311	0.406142	0.174152	0.036*	
C33	0.01886 (13)	0.51107 (10)	0.20646 (7)	0.0183 (2)	
H33A	−0.004918	0.431826	0.208581	0.022*	
H33B	−0.036645	0.525995	0.162206	0.022*	
C34	−0.02090 (13)	0.57483 (10)	0.26940 (7)	0.0184 (2)	
H34A	−0.007775	0.653382	0.264429	0.022*	
H34B	−0.122097	0.549385	0.270788	0.022*	
C35	0.22129 (14)	0.59142 (10)	0.33424 (7)	0.0190 (3)	
H35A	0.278875	0.578969	0.378650	0.023*	
H35B	0.243530	0.670137	0.330308	0.023*	
C36	0.25860 (13)	0.52464 (10)	0.27164 (6)	0.0179 (2)	
H36A	0.360404	0.546922	0.270211	0.021*	
H36B	0.241189	0.446177	0.276780	0.021*	
O7	0.52493 (11)	0.19182 (8)	0.42897 (5)	0.0269 (2)	
O8	0.47013 (12)	0.33271 (8)	0.37645 (5)	0.0287 (2)	
O9	0.19434 (11)	0.57629 (8)	0.50908 (5)	0.0230 (2)	
O10	0.17782 (10)	0.55155 (8)	0.62052 (5)	0.0234 (2)	
N7	0.42844 (12)	0.31409 (9)	0.61737 (6)	0.0198 (2)	
C40	0.46609 (13)	0.28029 (10)	0.55558 (7)	0.0189 (2)	
H40	0.519316	0.222923	0.554081	0.023*	
C41	0.43164 (13)	0.32440 (10)	0.49402 (7)	0.0180 (2)	
C42	0.35243 (13)	0.40754 (10)	0.49574 (7)	0.0181 (2)	
H42	0.327874	0.440131	0.454631	0.022*	
C43	0.30968 (13)	0.44226 (10)	0.55855 (7)	0.0178 (2)	
C44	0.35107 (14)	0.39327 (10)	0.61772 (7)	0.0191 (3)	
H44	0.322627	0.417573	0.660740	0.023*	
C45	0.47909 (14)	0.28018 (10)	0.42722 (7)	0.0214 (3)	
C46	0.22116 (13)	0.52903 (10)	0.56630 (7)	0.0183 (2)	
O11	0.02791 (10)	0.72051 (7)	0.52760 (5)	0.02092 (19)	
O12	0.01164 (11)	0.73611 (8)	0.41277 (5)	0.0239 (2)	
O13	−0.34905 (11)	1.07996 (8)	0.60863 (5)	0.0251 (2)	
O14	−0.21681 (11)	0.98202 (8)	0.67231 (5)	0.0238 (2)	
N8	−0.24408 (12)	0.96650 (9)	0.42223 (6)	0.0205 (2)	
C50	−0.16020 (14)	0.89128 (10)	0.42121 (7)	0.0187 (2)	
H50	−0.137927	0.864803	0.377035	0.022*	
C51	−0.10355 (13)	0.84945 (9)	0.48116 (6)	0.0163 (2)	
C52	−0.13637 (13)	0.88881 (10)	0.54549 (7)	0.0175 (2)	
H52	−0.099803	0.862807	0.587608	0.021*	
C53	−0.22352 (13)	0.96686 (10)	0.54757 (7)	0.0174 (2)	
C54	−0.27473 (14)	1.00335 (10)	0.48487 (7)	0.0198 (3)	
H54	−0.334015	1.056701	0.486515	0.024*	
C55	−0.01369 (13)	0.76158 (10)	0.47301 (6)	0.0168 (2)	
C56	−0.26179 (14)	1.00938 (10)	0.61586 (7)	0.0190 (3)	
O1W	0.24155 (13)	0.22831 (9)	0.27960 (6)	0.0337 (2)	
O2W	0.07059 (12)	0.34373 (8)	0.35168 (6)	0.0290 (2)	
O3W	−0.44895 (12)	1.18574 (8)	0.70984 (6)	0.0258 (2)	
H2	0.906 (2)	−0.2189 (18)	−0.1516 (11)	0.049 (6)*	
H3A	0.6603 (19)	0.0414 (15)	0.3468 (9)	0.030 (4)*	
H3B	0.597 (2)	0.1427 (16)	0.3624 (11)	0.040 (5)*	
H5A	0.368 (3)	0.352 (2)	−0.1569 (12)	0.060 (7)*	
H6A	0.0518 (18)	0.4917 (15)	0.3449 (9)	0.026 (4)*	
H6B	0.045 (2)	0.6073 (16)	0.3728 (10)	0.035 (5)*	
H9	0.136 (2)	0.6294 (19)	0.5180 (12)	0.056 (6)*	
H13	−0.368 (2)	1.1088 (19)	0.6518 (12)	0.055 (6)*	
H1W1	0.325 (2)	0.266 (2)	0.3110 (12)	0.073 (8)*	
H2W1	0.229 (3)	0.1601 (15)	0.2913 (13)	0.067 (7)*	
H1W2	0.126 (2)	0.3056 (18)	0.3362 (11)	0.048 (6)*	
H2W2	0.052 (2)	0.3183 (17)	0.3905 (12)	0.044 (5)*	
H1W3	−0.494 (2)	1.2315 (18)	0.6894 (11)	0.044 (6)*	
H2W3	−0.435 (2)	1.2139 (19)	0.7522 (13)	0.052 (6)*	

### 4-(3-Carboxy-1-ethyl-6-fluoro-4-oxo-1,4-dihydroquinolin-7-yl)piperazin-1-ium 5-carboxy-pyridine-3-carboxylate trihydrate (1) Atomic displacement parameters (Å^2^)

**Table:**

	*U*^11^	*U*^22^	*U*^33^	*U*^12^	*U*^13^	*U*^23^
F1	0.0179 (4)	0.0264 (4)	0.0218 (4)	0.0107 (3)	0.0017 (3)	−0.0036 (3)
O1	0.0192 (4)	0.0188 (4)	0.0171 (4)	0.0045 (3)	0.0029 (3)	−0.0005 (3)
O2	0.0228 (5)	0.0186 (4)	0.0178 (4)	0.0060 (4)	0.0047 (4)	−0.0005 (3)
O3	0.0229 (5)	0.0251 (5)	0.0234 (5)	0.0107 (4)	0.0063 (4)	−0.0007 (4)
N1	0.0177 (5)	0.0161 (5)	0.0163 (5)	0.0050 (4)	0.0042 (4)	0.0015 (4)
N2	0.0159 (5)	0.0170 (5)	0.0174 (5)	0.0019 (4)	0.0053 (4)	−0.0010 (4)
N3	0.0253 (6)	0.0187 (5)	0.0178 (5)	0.0100 (4)	0.0087 (4)	0.0043 (4)
C1	0.0187 (6)	0.0133 (5)	0.0207 (6)	0.0044 (4)	0.0075 (5)	0.0029 (5)
C2	0.0190 (6)	0.0128 (5)	0.0192 (6)	0.0032 (5)	0.0066 (5)	0.0020 (5)
C3	0.0188 (6)	0.0115 (5)	0.0171 (6)	0.0003 (4)	0.0048 (5)	0.0018 (4)
C4	0.0159 (6)	0.0126 (5)	0.0190 (6)	0.0017 (4)	0.0054 (5)	0.0018 (4)
C5	0.0160 (6)	0.0158 (6)	0.0180 (6)	0.0020 (5)	0.0025 (5)	0.0006 (5)
C6	0.0143 (6)	0.0160 (6)	0.0219 (6)	0.0049 (5)	0.0039 (5)	0.0009 (5)
C7	0.0190 (6)	0.0127 (5)	0.0173 (6)	0.0017 (4)	0.0064 (5)	0.0004 (4)
C8	0.0180 (6)	0.0149 (5)	0.0170 (6)	0.0025 (5)	0.0039 (5)	0.0022 (4)
C9	0.0171 (6)	0.0117 (5)	0.0199 (6)	0.0026 (4)	0.0061 (5)	0.0031 (4)
C10	0.0190 (6)	0.0137 (5)	0.0198 (6)	0.0019 (5)	0.0058 (5)	0.0014 (5)
C11	0.0181 (6)	0.0219 (6)	0.0188 (6)	0.0081 (5)	0.0009 (5)	−0.0014 (5)
C12	0.0367 (8)	0.0189 (6)	0.0192 (6)	0.0095 (6)	0.0015 (5)	0.0008 (5)
C13	0.0206 (6)	0.0194 (6)	0.0225 (6)	0.0002 (5)	0.0088 (5)	−0.0007 (5)
C14	0.0195 (6)	0.0217 (6)	0.0215 (6)	0.0057 (5)	0.0079 (5)	0.0033 (5)
C15	0.0232 (6)	0.0188 (6)	0.0193 (6)	0.0047 (5)	0.0049 (5)	0.0000 (5)
C16	0.0193 (6)	0.0196 (6)	0.0174 (6)	0.0047 (5)	0.0036 (5)	0.0005 (5)
F2	0.0241 (4)	0.0205 (4)	0.0217 (4)	0.0124 (3)	0.0059 (3)	0.0029 (3)
O4	0.0371 (6)	0.0321 (5)	0.0179 (5)	0.0153 (4)	0.0084 (4)	0.0050 (4)
O5	0.0381 (6)	0.0367 (6)	0.0231 (5)	0.0131 (5)	0.0136 (4)	−0.0002 (4)
O6	0.0249 (5)	0.0316 (5)	0.0360 (6)	0.0085 (4)	0.0098 (4)	−0.0099 (4)
N4	0.0176 (5)	0.0144 (5)	0.0206 (5)	0.0043 (4)	0.0044 (4)	0.0011 (4)
N5	0.0162 (5)	0.0165 (5)	0.0169 (5)	0.0040 (4)	0.0049 (4)	0.0013 (4)
N6	0.0207 (5)	0.0156 (5)	0.0183 (5)	0.0038 (4)	0.0076 (4)	0.0019 (4)
C21	0.0171 (6)	0.0148 (6)	0.0258 (6)	0.0027 (5)	0.0069 (5)	−0.0018 (5)
C22	0.0198 (6)	0.0156 (6)	0.0226 (6)	0.0015 (5)	0.0077 (5)	−0.0016 (5)
C23	0.0214 (6)	0.0161 (6)	0.0209 (6)	0.0013 (5)	0.0067 (5)	0.0011 (5)
C24	0.0190 (6)	0.0140 (5)	0.0187 (6)	0.0016 (5)	0.0053 (5)	0.0008 (5)
C25	0.0199 (6)	0.0166 (6)	0.0184 (6)	0.0040 (5)	0.0035 (5)	0.0025 (5)
C26	0.0169 (6)	0.0132 (5)	0.0213 (6)	0.0052 (5)	0.0039 (5)	0.0015 (5)
C27	0.0162 (6)	0.0132 (5)	0.0181 (6)	0.0006 (4)	0.0050 (5)	0.0001 (4)
C28	0.0184 (6)	0.0151 (5)	0.0181 (6)	0.0029 (5)	0.0046 (5)	0.0027 (5)
C29	0.0160 (6)	0.0117 (5)	0.0217 (6)	0.0020 (4)	0.0043 (5)	0.0009 (4)
C30	0.0215 (7)	0.0202 (6)	0.0270 (7)	0.0002 (5)	0.0093 (5)	−0.0048 (5)
C31	0.0218 (6)	0.0176 (6)	0.0230 (6)	0.0090 (5)	0.0039 (5)	0.0038 (5)
C32	0.0240 (7)	0.0230 (6)	0.0237 (6)	0.0072 (5)	0.0012 (5)	0.0007 (5)
C33	0.0165 (6)	0.0182 (6)	0.0198 (6)	0.0026 (5)	0.0055 (5)	−0.0006 (5)
C34	0.0188 (6)	0.0180 (6)	0.0197 (6)	0.0059 (5)	0.0056 (5)	0.0020 (5)
C35	0.0188 (6)	0.0189 (6)	0.0183 (6)	0.0013 (5)	0.0043 (5)	0.0002 (5)
C36	0.0181 (6)	0.0183 (6)	0.0180 (6)	0.0046 (5)	0.0046 (5)	0.0023 (5)
O7	0.0351 (6)	0.0244 (5)	0.0257 (5)	0.0125 (4)	0.0137 (4)	0.0014 (4)
O8	0.0378 (6)	0.0256 (5)	0.0249 (5)	0.0073 (4)	0.0106 (4)	0.0041 (4)
O9	0.0297 (5)	0.0211 (4)	0.0225 (5)	0.0126 (4)	0.0087 (4)	0.0048 (4)
O10	0.0271 (5)	0.0228 (5)	0.0254 (5)	0.0103 (4)	0.0125 (4)	0.0049 (4)
N7	0.0208 (5)	0.0179 (5)	0.0214 (5)	0.0048 (4)	0.0049 (4)	0.0017 (4)
C40	0.0179 (6)	0.0152 (6)	0.0235 (6)	0.0032 (5)	0.0054 (5)	−0.0002 (5)
C41	0.0175 (6)	0.0147 (5)	0.0209 (6)	0.0002 (5)	0.0053 (5)	−0.0004 (5)
C42	0.0183 (6)	0.0146 (5)	0.0206 (6)	0.0013 (5)	0.0040 (5)	0.0016 (5)
C43	0.0161 (6)	0.0141 (5)	0.0226 (6)	0.0014 (5)	0.0042 (5)	0.0011 (5)
C44	0.0195 (6)	0.0172 (6)	0.0207 (6)	0.0032 (5)	0.0062 (5)	0.0000 (5)
C45	0.0243 (7)	0.0170 (6)	0.0231 (6)	0.0025 (5)	0.0083 (5)	0.0003 (5)
C46	0.0175 (6)	0.0152 (6)	0.0225 (6)	0.0025 (5)	0.0052 (5)	0.0018 (5)
O11	0.0265 (5)	0.0207 (4)	0.0188 (4)	0.0116 (4)	0.0059 (4)	0.0039 (3)
O12	0.0342 (5)	0.0228 (5)	0.0183 (4)	0.0133 (4)	0.0089 (4)	0.0011 (4)
O13	0.0329 (5)	0.0253 (5)	0.0232 (5)	0.0175 (4)	0.0107 (4)	0.0038 (4)
O14	0.0321 (5)	0.0223 (5)	0.0192 (4)	0.0106 (4)	0.0070 (4)	0.0016 (4)
N8	0.0246 (6)	0.0183 (5)	0.0204 (5)	0.0077 (4)	0.0055 (4)	0.0037 (4)
C50	0.0215 (6)	0.0161 (6)	0.0194 (6)	0.0046 (5)	0.0059 (5)	0.0015 (5)
C51	0.0175 (6)	0.0117 (5)	0.0194 (6)	0.0017 (5)	0.0045 (5)	0.0008 (5)
C52	0.0192 (6)	0.0143 (5)	0.0188 (6)	0.0026 (5)	0.0033 (5)	0.0020 (5)
C53	0.0192 (6)	0.0136 (5)	0.0198 (6)	0.0034 (5)	0.0054 (5)	0.0005 (5)
C54	0.0220 (6)	0.0163 (6)	0.0232 (6)	0.0065 (5)	0.0068 (5)	0.0027 (5)
C55	0.0168 (6)	0.0137 (5)	0.0194 (6)	0.0025 (5)	0.0037 (5)	0.0003 (4)
C56	0.0215 (6)	0.0145 (5)	0.0216 (6)	0.0040 (5)	0.0061 (5)	0.0013 (5)
O1W	0.0412 (6)	0.0317 (6)	0.0294 (5)	0.0156 (5)	0.0021 (5)	0.0028 (4)
O2W	0.0377 (6)	0.0224 (5)	0.0349 (6)	0.0117 (4)	0.0194 (5)	0.0099 (4)
O3W	0.0363 (6)	0.0235 (5)	0.0232 (5)	0.0143 (4)	0.0122 (4)	0.0050 (4)

### 4-(3-Carboxy-1-ethyl-6-fluoro-4-oxo-1,4-dihydroquinolin-7-yl)piperazin-1-ium 5-carboxy-pyridine-3-carboxylate trihydrate (1) Geometric parameters (Å, º)

**Table:**

F1—C6	1.3549 (14)	C23—C24	1.4505 (17)
O1—C3	1.2622 (15)	C24—C29	1.4064 (17)
O2—C10	1.3331 (16)	C24—C25	1.4078 (18)
O2—H2	0.97 (2)	C25—C26	1.3604 (18)
O3—C10	1.2160 (16)	C25—H25	0.9500
N1—C1	1.3404 (16)	C26—C27	1.4159 (17)
N1—C9	1.3933 (16)	C27—C28	1.3853 (18)
N1—C11	1.4832 (16)	C28—C29	1.4058 (17)
N2—C7	1.4074 (15)	C28—H28	0.9500
N2—C16	1.4664 (16)	C31—C32	1.5233 (18)
N2—C13	1.4798 (16)	C31—H31A	0.9900
N3—C14	1.4808 (17)	C31—H31B	0.9900
N3—C15	1.4864 (17)	C32—H32A	0.9800
N3—H3A	0.945 (19)	C32—H32B	0.9800
N3—H3B	0.94 (2)	C32—H32C	0.9800
C1—C2	1.3756 (18)	C33—C34	1.5132 (16)
C1—H1	0.9500	C33—H33A	0.9900
C2—C3	1.4285 (18)	C33—H33B	0.9900
C2—C10	1.4828 (17)	C34—H34A	0.9900
C3—C4	1.4547 (17)	C34—H34B	0.9900
C4—C5	1.4047 (18)	C35—C36	1.5153 (17)
C4—C9	1.4053 (17)	C35—H35A	0.9900
C5—C6	1.3574 (17)	C35—H35B	0.9900
C5—H5	0.9500	C36—H36A	0.9900
C6—C7	1.4157 (17)	C36—H36B	0.9900
C7—C8	1.3818 (18)	O7—C45	1.2589 (17)
C8—C9	1.4086 (17)	O8—C45	1.2442 (17)
C8—H8	0.9500	O9—C46	1.3245 (16)
C11—C12	1.5167 (19)	O9—H9	0.95 (2)
C11—H11A	0.9900	O10—C46	1.2113 (16)
C11—H11B	0.9900	N7—C44	1.3362 (17)
C12—H12A	0.9800	N7—C40	1.3463 (17)
C12—H12B	0.9800	C40—C41	1.3861 (18)
C12—H12C	0.9800	C40—H40	0.9500
C13—C14	1.5105 (17)	C41—C42	1.3900 (18)
C13—H13A	0.9900	C41—C45	1.5143 (17)
C13—H13B	0.9900	C42—C43	1.3904 (18)
C14—H14A	0.9900	C42—H42	0.9500
C14—H14B	0.9900	C43—C44	1.3945 (18)
C15—C16	1.5178 (17)	C43—C46	1.4978 (17)
C15—H15A	0.9900	C44—H44	0.9500
C15—H15B	0.9900	O11—C55	1.2649 (15)
C16—H16A	0.9900	O12—C55	1.2451 (15)
C16—H16B	0.9900	O13—C56	1.3225 (16)
F2—C26	1.3546 (14)	O13—H13	0.93 (2)
O4—C23	1.2696 (16)	O14—C56	1.2180 (16)
O5—C30	1.3265 (18)	N8—C50	1.3405 (17)
O5—H5A	0.98 (3)	N8—C54	1.3409 (17)
O6—C30	1.2156 (18)	C50—C51	1.3972 (17)
N4—C21	1.3390 (17)	C50—H50	0.9500
N4—C29	1.3969 (16)	C51—C52	1.3880 (17)
N4—C31	1.4852 (16)	C51—C55	1.5188 (17)
N5—C27	1.4037 (15)	C52—C53	1.3924 (18)
N5—C36	1.4669 (16)	C52—H52	0.9500
N5—C33	1.4821 (16)	C53—C54	1.3916 (18)
N6—C35	1.4854 (16)	C53—C56	1.4907 (17)
N6—C34	1.4886 (16)	C54—H54	0.9500
N6—H6A	0.887 (18)	O1W—H1W1	0.947 (17)
N6—H6B	0.94 (2)	O1W—H2W1	0.904 (16)
C21—C22	1.3808 (19)	O2W—H1W2	0.84 (2)
C21—H21	0.9500	O2W—H2W2	0.88 (2)
C22—C23	1.4230 (18)	O3W—H1W3	0.87 (2)
C22—C30	1.4872 (18)	O3W—H2W3	0.83 (3)
			
C10—O2—H2	106.3 (12)	C26—C25—C24	119.37 (12)
C1—N1—C9	120.08 (11)	C26—C25—H25	120.3
C1—N1—C11	119.43 (10)	C24—C25—H25	120.3
C9—N1—C11	120.42 (10)	F2—C26—C25	118.56 (11)
C7—N2—C16	115.79 (10)	F2—C26—C27	117.99 (11)
C7—N2—C13	111.44 (10)	C25—C26—C27	123.42 (11)
C16—N2—C13	110.95 (10)	C28—C27—N5	122.92 (11)
C14—N3—C15	111.06 (10)	C28—C27—C26	116.80 (11)
C14—N3—H3A	111.4 (11)	N5—C27—C26	120.18 (11)
C15—N3—H3A	105.5 (11)	C27—C28—C29	121.34 (11)
C14—N3—H3B	109.8 (12)	C27—C28—H28	119.3
C15—N3—H3B	111.8 (12)	C29—C28—H28	119.3
H3A—N3—H3B	107.2 (16)	N4—C29—C28	120.50 (11)
N1—C1—C2	123.61 (12)	N4—C29—C24	119.47 (11)
N1—C1—H1	118.2	C28—C29—C24	120.03 (11)
C2—C1—H1	118.2	O6—C30—O5	120.97 (12)
C1—C2—C3	120.46 (11)	O6—C30—C22	124.20 (13)
C1—C2—C10	117.87 (11)	O5—C30—C22	114.83 (12)
C3—C2—C10	121.59 (11)	N4—C31—C32	112.61 (10)
O1—C3—C2	123.68 (11)	N4—C31—H31A	109.1
O1—C3—C4	121.19 (11)	C32—C31—H31A	109.1
C2—C3—C4	115.13 (11)	N4—C31—H31B	109.1
C5—C4—C9	118.65 (11)	C32—C31—H31B	109.1
C5—C4—C3	119.63 (11)	H31A—C31—H31B	107.8
C9—C4—C3	121.67 (11)	C31—C32—H32A	109.5
C6—C5—C4	119.54 (11)	C31—C32—H32B	109.5
C6—C5—H5	120.2	H32A—C32—H32B	109.5
C4—C5—H5	120.2	C31—C32—H32C	109.5
F1—C6—C5	119.21 (11)	H32A—C32—H32C	109.5
F1—C6—C7	117.41 (11)	H32B—C32—H32C	109.5
C5—C6—C7	123.34 (12)	N5—C33—C34	110.95 (10)
C8—C7—N2	124.24 (11)	N5—C33—H33A	109.4
C8—C7—C6	117.05 (11)	C34—C33—H33A	109.4
N2—C7—C6	118.70 (11)	N5—C33—H33B	109.4
C7—C8—C9	120.82 (11)	C34—C33—H33B	109.4
C7—C8—H8	119.6	H33A—C33—H33B	108.0
C9—C8—H8	119.6	N6—C34—C33	110.65 (10)
N1—C9—C4	118.97 (11)	N6—C34—H34A	109.5
N1—C9—C8	120.60 (11)	C33—C34—H34A	109.5
C4—C9—C8	120.43 (11)	N6—C34—H34B	109.5
O3—C10—O2	121.26 (11)	C33—C34—H34B	109.5
O3—C10—C2	123.26 (12)	H34A—C34—H34B	108.1
O2—C10—C2	115.48 (11)	N6—C35—C36	110.39 (10)
N1—C11—C12	111.19 (11)	N6—C35—H35A	109.6
N1—C11—H11A	109.4	C36—C35—H35A	109.6
C12—C11—H11A	109.4	N6—C35—H35B	109.6
N1—C11—H11B	109.4	C36—C35—H35B	109.6
C12—C11—H11B	109.4	H35A—C35—H35B	108.1
H11A—C11—H11B	108.0	N5—C36—C35	109.91 (10)
C11—C12—H12A	109.5	N5—C36—H36A	109.7
C11—C12—H12B	109.5	C35—C36—H36A	109.7
H12A—C12—H12B	109.5	N5—C36—H36B	109.7
C11—C12—H12C	109.5	C35—C36—H36B	109.7
H12A—C12—H12C	109.5	H36A—C36—H36B	108.2
H12B—C12—H12C	109.5	C46—O9—H9	109.0 (13)
N2—C13—C14	110.58 (10)	C44—N7—C40	116.97 (11)
N2—C13—H13A	109.5	N7—C40—C41	123.76 (12)
C14—C13—H13A	109.5	N7—C40—H40	118.1
N2—C13—H13B	109.5	C41—C40—H40	118.1
C14—C13—H13B	109.5	C40—C41—C42	118.34 (11)
H13A—C13—H13B	108.1	C40—C41—C45	119.51 (11)
N3—C14—C13	109.91 (11)	C42—C41—C45	122.15 (11)
N3—C14—H14A	109.7	C41—C42—C43	118.98 (12)
C13—C14—H14A	109.7	C41—C42—H42	120.5
N3—C14—H14B	109.7	C43—C42—H42	120.5
C13—C14—H14B	109.7	C42—C43—C44	118.20 (12)
H14A—C14—H14B	108.2	C42—C43—C46	123.77 (11)
N3—C15—C16	110.17 (10)	C44—C43—C46	118.03 (11)
N3—C15—H15A	109.6	N7—C44—C43	123.74 (12)
C16—C15—H15A	109.6	N7—C44—H44	118.1
N3—C15—H15B	109.6	C43—C44—H44	118.1
C16—C15—H15B	109.6	O8—C45—O7	126.06 (12)
H15A—C15—H15B	108.1	O8—C45—C41	118.52 (12)
N2—C16—C15	110.09 (10)	O7—C45—C41	115.42 (11)
N2—C16—H16A	109.6	O10—C46—O9	124.15 (12)
C15—C16—H16A	109.6	O10—C46—C43	121.68 (12)
N2—C16—H16B	109.6	O9—C46—C43	114.16 (11)
C15—C16—H16B	109.6	C56—O13—H13	112.3 (14)
H16A—C16—H16B	108.2	C50—N8—C54	117.50 (11)
C30—O5—H5A	106.1 (14)	N8—C50—C51	123.98 (12)
C21—N4—C29	120.05 (11)	N8—C50—H50	118.0
C21—N4—C31	119.69 (11)	C51—C50—H50	118.0
C29—N4—C31	120.25 (10)	C52—C51—C50	117.61 (11)
C27—N5—C36	115.34 (10)	C52—C51—C55	123.16 (11)
C27—N5—C33	114.99 (10)	C50—C51—C55	119.19 (11)
C36—N5—C33	111.25 (9)	C51—C52—C53	119.22 (11)
C35—N6—C34	110.73 (9)	C51—C52—H52	120.4
C35—N6—H6A	108.6 (11)	C53—C52—H52	120.4
C34—N6—H6A	111.7 (11)	C54—C53—C52	118.81 (11)
C35—N6—H6B	109.2 (11)	C54—C53—C56	120.95 (11)
C34—N6—H6B	106.4 (11)	C52—C53—C56	120.24 (11)
H6A—N6—H6B	110.2 (16)	N8—C54—C53	122.89 (12)
N4—C21—C22	123.25 (12)	N8—C54—H54	118.6
N4—C21—H21	118.4	C53—C54—H54	118.6
C22—C21—H21	118.4	O12—C55—O11	125.90 (12)
C21—C22—C23	120.32 (11)	O12—C55—C51	116.70 (11)
C21—C22—C30	119.10 (12)	O11—C55—C51	117.40 (11)
C23—C22—C30	120.57 (12)	O14—C56—O13	123.72 (12)
O4—C23—C22	122.72 (12)	O14—C56—C53	123.35 (12)
O4—C23—C24	121.21 (12)	O13—C56—C53	112.93 (11)
C22—C23—C24	116.07 (11)	H1W1—O1W—H2W1	104 (2)
C29—C24—C25	118.92 (11)	H1W2—O2W—H2W2	105.7 (19)
C29—C24—C23	120.71 (12)	H1W3—O3W—H2W3	101 (2)
C25—C24—C23	120.37 (11)		
			
C9—N1—C1—C2	2.19 (18)	C36—N5—C27—C26	−163.87 (11)
C11—N1—C1—C2	179.19 (11)	C33—N5—C27—C26	64.60 (14)
N1—C1—C2—C3	0.47 (18)	F2—C26—C27—C28	−176.02 (10)
N1—C1—C2—C10	177.09 (11)	C25—C26—C27—C28	2.07 (18)
C1—C2—C3—O1	176.79 (11)	F2—C26—C27—N5	0.35 (17)
C10—C2—C3—O1	0.30 (18)	C25—C26—C27—N5	178.44 (11)
C1—C2—C3—C4	−2.58 (16)	N5—C27—C28—C29	−176.19 (11)
C10—C2—C3—C4	−179.07 (10)	C26—C27—C28—C29	0.07 (17)
O1—C3—C4—C5	0.25 (17)	C21—N4—C29—C28	179.09 (11)
C2—C3—C4—C5	179.64 (10)	C31—N4—C29—C28	−1.05 (17)
O1—C3—C4—C9	−177.16 (11)	C21—N4—C29—C24	−0.29 (17)
C2—C3—C4—C9	2.23 (16)	C31—N4—C29—C24	179.57 (11)
C9—C4—C5—C6	−2.20 (17)	C27—C28—C29—N4	177.62 (11)
C3—C4—C5—C6	−179.68 (11)	C27—C28—C29—C24	−3.00 (18)
C4—C5—C6—F1	176.11 (10)	C25—C24—C29—N4	−176.75 (11)
C4—C5—C6—C7	−1.52 (19)	C23—C24—C29—N4	3.26 (17)
C16—N2—C7—C8	12.22 (17)	C25—C24—C29—C28	3.87 (17)
C13—N2—C7—C8	−115.84 (13)	C23—C24—C29—C28	−176.12 (11)
C16—N2—C7—C6	−167.46 (11)	C21—C22—C30—O6	−0.8 (2)
C13—N2—C7—C6	64.49 (14)	C23—C22—C30—O6	179.25 (12)
F1—C6—C7—C8	−173.54 (10)	C21—C22—C30—O5	179.12 (11)
C5—C6—C7—C8	4.14 (18)	C23—C22—C30—O5	−0.81 (18)
F1—C6—C7—N2	6.16 (16)	C21—N4—C31—C32	105.85 (13)
C5—C6—C7—N2	−176.16 (11)	C29—N4—C31—C32	−74.01 (14)
N2—C7—C8—C9	177.31 (10)	C27—N5—C33—C34	−169.65 (10)
C6—C7—C8—C9	−3.01 (17)	C36—N5—C33—C34	56.90 (13)
C1—N1—C9—C4	−2.47 (17)	C35—N6—C34—C33	55.74 (13)
C11—N1—C9—C4	−179.44 (10)	N5—C33—C34—N6	−55.09 (13)
C1—N1—C9—C8	177.09 (10)	C34—N6—C35—C36	−57.47 (13)
C11—N1—C9—C8	0.13 (17)	C27—N5—C36—C35	168.53 (10)
C5—C4—C9—N1	−177.22 (10)	C33—N5—C36—C35	−58.19 (12)
C3—C4—C9—N1	0.21 (17)	N6—C35—C36—N5	58.46 (13)
C5—C4—C9—C8	3.22 (17)	C44—N7—C40—C41	1.38 (18)
C3—C4—C9—C8	−179.35 (10)	N7—C40—C41—C42	−0.70 (19)
C7—C8—C9—N1	179.90 (11)	N7—C40—C41—C45	−179.89 (11)
C7—C8—C9—C4	−0.55 (17)	C40—C41—C42—C43	−0.70 (18)
C1—C2—C10—O3	4.32 (18)	C45—C41—C42—C43	178.47 (11)
C3—C2—C10—O3	−179.10 (11)	C41—C42—C43—C44	1.32 (18)
C1—C2—C10—O2	−174.93 (10)	C41—C42—C43—C46	−178.43 (11)
C3—C2—C10—O2	1.65 (16)	C40—N7—C44—C43	−0.70 (18)
C1—N1—C11—C12	−99.65 (13)	C42—C43—C44—N7	−0.63 (19)
C9—N1—C11—C12	77.34 (14)	C46—C43—C44—N7	179.13 (11)
C7—N2—C13—C14	−171.06 (11)	C40—C41—C45—O8	−167.22 (12)
C16—N2—C13—C14	58.32 (14)	C42—C41—C45—O8	13.62 (19)
C15—N3—C14—C13	57.01 (13)	C40—C41—C45—O7	12.66 (18)
N2—C13—C14—N3	−57.07 (14)	C42—C41—C45—O7	−166.50 (12)
C14—N3—C15—C16	−57.21 (13)	C42—C43—C46—O10	174.72 (12)
C7—N2—C16—C15	173.61 (10)	C44—C43—C46—O10	−5.02 (18)
C13—N2—C16—C15	−58.08 (13)	C42—C43—C46—O9	−4.47 (18)
N3—C15—C16—N2	57.27 (13)	C44—C43—C46—O9	175.79 (11)
C29—N4—C21—C22	−1.89 (18)	C54—N8—C50—C51	0.05 (19)
C31—N4—C21—C22	178.25 (11)	N8—C50—C51—C52	−0.16 (19)
N4—C21—C22—C23	1.00 (19)	N8—C50—C51—C55	178.00 (11)
N4—C21—C22—C30	−178.93 (11)	C50—C51—C52—C53	0.24 (18)
C21—C22—C23—O4	−178.25 (12)	C55—C51—C52—C53	−177.83 (11)
C30—C22—C23—O4	1.68 (19)	C51—C52—C53—C54	−0.23 (18)
C21—C22—C23—C24	1.91 (17)	C51—C52—C53—C56	179.23 (11)
C30—C22—C23—C24	−178.17 (11)	C50—N8—C54—C53	−0.03 (19)
O4—C23—C24—C29	176.17 (12)	C52—C53—C54—N8	0.12 (19)
C22—C23—C24—C29	−3.98 (17)	C56—C53—C54—N8	−179.34 (11)
O4—C23—C24—C25	−3.82 (19)	C52—C51—C55—O12	−177.75 (11)
C22—C23—C24—C25	176.02 (11)	C50—C51—C55—O12	4.20 (17)
C29—C24—C25—C26	−1.83 (18)	C52—C51—C55—O11	2.64 (17)
C23—C24—C25—C26	178.17 (11)	C50—C51—C55—O11	−175.40 (11)
C24—C25—C26—F2	176.90 (10)	C54—C53—C56—O14	−178.09 (12)
C24—C25—C26—C27	−1.18 (19)	C52—C53—C56—O14	2.46 (19)
C36—N5—C27—C28	12.27 (16)	C54—C53—C56—O13	1.69 (17)
C33—N5—C27—C28	−119.26 (13)	C52—C53—C56—O13	−177.76 (11)

### 4-(3-Carboxy-1-ethyl-6-fluoro-4-oxo-1,4-dihydroquinolin-7-yl)piperazin-1-ium 5-carboxy-pyridine-3-carboxylate trihydrate (1) Hydrogen-bond geometry (Å, º)

**Table:**

*D*—H···*A*	*D*—H	H···*A*	*D*···*A*	*D*—H···*A*
O2—H2···O1	0.97 (2)	1.64 (2)	2.5554 (13)	156 (2)
O5—H5*A*···O4	0.98 (3)	1.54 (3)	2.4830 (14)	158 (2)
O9—H9···O11	0.95 (2)	1.69 (3)	2.6396 (13)	176 (2)
O13—H13···O3*W*	0.93 (2)	1.75 (2)	2.6342 (14)	157 (2)
N3—H3*B*···O7	0.94 (2)	1.66 (2)	2.5925 (14)	170 (2)
N3—H3*A*···N8^i^	0.945 (19)	2.009 (19)	2.8825 (16)	153 (2)
N6—H6*A*···O2*W*	0.887 (18)	1.908 (19)	2.7646 (15)	162 (2)
N6—H6*B*···O12	0.94 (2)	1.81 (2)	2.6856 (14)	154 (2)
C5—H5···F1^ii^	0.95	2.32	3.0193 (15)	130
C11—H11*B*···O6^iii^	0.99	2.35	3.2564 (16)	152
C12—H12*B*···F2^i^	0.98	2.36	3.2576 (15)	151
C15—H15*A*···O11^iv^	0.99	2.38	3.3290 (16)	161
C33—H33*A*···O2^ii^	0.99	2.36	3.2282 (15)	145
O1*W*—H1*W*1···O8	0.95 (2)	1.76 (2)	2.7047 (2)	174 (2)
O1*W*—H2*W*1···O14^v^	0.90 (2)	1.98 (2)	2.8834 (2)	173 (2)
O2*W*—H1*W*2···O1*W*	0.84 (2)	1.97 (2)	2.7974 (16)	167 (2)
O2*W*—H2*W*2···O11^v^	0.88 (2)	1.95 (2)	2.8171 (14)	166 (2)
O3*W*—H1*W*3···N7^vi^	0.87 (2)	1.96 (2)	2.7937 (15)	161 (2)
O3*W*—H2*W*3···O5^vii^	0.83 (3)	2.41 (2)	3.0846 (14)	138 (2)

Symmetry codes: (i) *x*+1, *y*−1, *z*; (ii) −*x*+1, −*y*, −*z*; (iii) −*x*+2, −*y*, −*z*; (iv) −*x*+1, −*y*+1, −*z*+1; (v) −*x*, −*y*+1, −*z*+1; (vi) *x*−1, *y*+1, *z*; (vii) *x*−1, *y*+1, *z*+1.

### 4-(3-Carboxy-1-ethyl-6-fluoro-4-oxo-1,4-dihydroquinolin-7-yl)piperazin-1-ium pyridazine-3-carboxylate (2) Crystal data

**Table:**

C_16_H_19_FN_3_O_3_^+^·C_5_H_3_N_2_O_2_^−^	*Z* = 4
*M**_r_* = 443.43	*F*(000) = 928
Triclinic, *P*1	*D*_x_ = 1.466 Mg m^−^^3^
*a* = 9.0971 (1) Å	Cu *K*α radiation, λ = 1.54184 Å
*b* = 12.9566 (1) Å	Cell parameters from 34780 reflections
*c* = 19.2412 (2) Å	θ = 3.8–77.3°
α = 104.666 (1)°	µ = 0.95 mm^−^^1^
β = 95.813 (1)°	*T* = 100 K
γ = 110.485 (1)°	Plate, colourless
*V* = 2009.65 (4) Å^3^	0.25 × 0.19 × 0.05 mm

### 4-(3-Carboxy-1-ethyl-6-fluoro-4-oxo-1,4-dihydroquinolin-7-yl)piperazin-1-ium pyridazine-3-carboxylate (2) Data collection

**Table:**

Rigaku, XtaLAB Synergy, Dualflex, HyPix diffractometer	8113 independent reflections
Radiation source: micro-focus sealed X-ray tube, PhotonJet (Cu) X-ray Source	7634 reflections with *I* > 2σ(*I*)
Mirror monochromator	*R*_int_ = 0.019
Detector resolution: 10.0000 pixels mm^-1^	θ_max_ = 77.4°, θ_min_ = 3.8°
ω scans	*h* = −10→11
Absorption correction: gaussian (CrysAlisPro; Rigaku OD, 2025)	*k* = −15→16
*T*_min_ = 0.626, *T*_max_ = 1.000	*l* = −24→23
45406 measured reflections	

### 4-(3-Carboxy-1-ethyl-6-fluoro-4-oxo-1,4-dihydroquinolin-7-yl)piperazin-1-ium pyridazine-3-carboxylate (2) Refinement

**Table:**

Refinement on *F*^2^	Primary atom site location: structure-invariant direct methods
Least-squares matrix: full	Hydrogen site location: mixed
*R*[*F*^2^ > 2σ(*F*^2^)] = 0.031	H atoms treated by a mixture of independent and constrained refinement
*wR*(*F*^2^) = 0.084	*w* = 1/[σ^2^(*F*_o_^2^) + (0.0428*P*)^2^ + 0.6879*P*] where *P* = (*F*_o_^2^ + 2*F*_c_^2^)/3
*S* = 1.05	(Δ/σ)_max_ = 0.001
8113 reflections	Δρ_max_ = 0.25 e Å^−^^3^
603 parameters	Δρ_min_ = −0.21 e Å^−^^3^
0 restraints	

### 4-(3-Carboxy-1-ethyl-6-fluoro-4-oxo-1,4-dihydroquinolin-7-yl)piperazin-1-ium pyridazine-3-carboxylate (2) Special details

**Table:**

Geometry. All esds (except the esd in the dihedral angle between two l.s. planes) are estimated using the full covariance matrix. The cell esds are taken into account individually in the estimation of esds in distances, angles and torsion angles; correlations between esds in cell parameters are only used when they are defined by crystal symmetry. An approximate (isotropic) treatment of cell esds is used for estimating esds involving l.s. planes.

### 4-(3-Carboxy-1-ethyl-6-fluoro-4-oxo-1,4-dihydroquinolin-7-yl)piperazin-1-ium pyridazine-3-carboxylate (2) Fractional atomic coordinates and isotropic or equivalent isotropic displacement parameters (Å^2^)

**Table:**

	*x*	*y*	*z*	*U*_iso_*/*U*_eq_	
F1	0.36929 (8)	0.56898 (5)	0.71179 (3)	0.02401 (14)	
O1	0.51080 (9)	0.36291 (6)	0.48658 (4)	0.02014 (16)	
O2	0.53287 (9)	0.20568 (7)	0.38541 (4)	0.02135 (16)	
O3	0.47224 (9)	0.02945 (6)	0.39598 (4)	0.02155 (16)	
N1	0.29377 (10)	0.11660 (7)	0.58316 (5)	0.01631 (17)	
N2	0.25725 (10)	0.41334 (7)	0.78774 (5)	0.01732 (17)	
N3	0.12634 (11)	0.44497 (8)	0.91723 (5)	0.0232 (2)	
C1	0.34766 (12)	0.08884 (9)	0.52180 (5)	0.01644 (19)	
H1	0.333827	0.010486	0.500777	0.020*	
C2	0.42220 (12)	0.16825 (9)	0.48760 (5)	0.0164 (2)	
C3	0.44332 (11)	0.28707 (9)	0.51632 (6)	0.0165 (2)	
C4	0.38257 (12)	0.31573 (9)	0.58243 (5)	0.01630 (19)	
C5	0.39718 (12)	0.43021 (9)	0.61530 (6)	0.0178 (2)	
H5	0.440187	0.488056	0.592529	0.021*	
C6	0.34937 (12)	0.45726 (8)	0.67973 (6)	0.0181 (2)	
C7	0.28665 (12)	0.37591 (9)	0.71729 (5)	0.0168 (2)	
C8	0.26614 (12)	0.26245 (9)	0.68323 (5)	0.0170 (2)	
H8	0.219683	0.204727	0.705623	0.020*	
C9	0.31273 (11)	0.23123 (8)	0.61624 (5)	0.01576 (19)	
C10	0.47810 (12)	0.12736 (9)	0.41999 (5)	0.0172 (2)	
C11	0.20953 (13)	0.02375 (9)	0.61409 (6)	0.0189 (2)	
H11A	0.251163	0.050424	0.667828	0.023*	
H11B	0.232630	−0.045391	0.592531	0.023*	
C12	0.02891 (13)	−0.00969 (9)	0.59900 (6)	0.0242 (2)	
H12A	−0.019484	−0.059436	0.628219	0.036*	
H12B	−0.016158	−0.051622	0.546671	0.036*	
H12C	0.005979	0.060435	0.612374	0.036*	
C13	0.12390 (12)	0.45322 (9)	0.79015 (6)	0.0201 (2)	
H13A	0.020357	0.385714	0.770054	0.024*	
H13B	0.132536	0.504891	0.759316	0.024*	
C14	0.12832 (13)	0.51772 (9)	0.86843 (6)	0.0233 (2)	
H14A	0.226588	0.589760	0.886579	0.028*	
H14B	0.034393	0.539528	0.869611	0.028*	
C15	0.25414 (13)	0.39901 (9)	0.91187 (6)	0.0225 (2)	
H15A	0.242311	0.345488	0.941576	0.027*	
H15B	0.360354	0.463788	0.932013	0.027*	
C16	0.24551 (13)	0.33513 (9)	0.83249 (6)	0.0194 (2)	
H16A	0.334427	0.307915	0.829811	0.023*	
H16B	0.142862	0.266632	0.813290	0.023*	
F2	1.19558 (7)	1.00676 (5)	0.77694 (3)	0.02172 (14)	
O4	1.03759 (9)	1.19786 (6)	0.99992 (4)	0.02192 (16)	
O5	0.89197 (9)	1.23291 (6)	1.10250 (4)	0.02316 (16)	
O6	0.66496 (9)	1.09502 (6)	1.10469 (4)	0.02197 (16)	
N4	0.68014 (10)	0.87336 (7)	0.90886 (5)	0.01628 (17)	
N5	0.95393 (10)	0.79482 (7)	0.70781 (5)	0.01697 (17)	
N6	0.96497 (11)	0.62052 (8)	0.58612 (5)	0.01960 (18)	
C21	0.67623 (12)	0.94555 (9)	0.97149 (5)	0.0169 (2)	
H21	0.589271	0.919297	0.994759	0.020*	
C22	0.79145 (12)	1.05599 (8)	1.00415 (5)	0.0165 (2)	
C23	0.92738 (12)	1.09775 (9)	0.97242 (5)	0.0169 (2)	
C24	0.93343 (12)	1.01687 (8)	0.90620 (5)	0.0163 (2)	
C25	1.06560 (12)	1.04859 (9)	0.87253 (5)	0.0171 (2)	
H25	1.151927	1.121916	0.893741	0.020*	
C26	1.06891 (12)	0.97381 (9)	0.80964 (6)	0.0171 (2)	
C27	0.94327 (12)	0.86402 (9)	0.77466 (5)	0.01634 (19)	
C28	0.81318 (12)	0.83293 (8)	0.80764 (5)	0.0168 (2)	
H28	0.726120	0.760302	0.785211	0.020*	
C29	0.80791 (12)	0.90726 (8)	0.87388 (5)	0.01571 (19)	
C30	0.77508 (12)	1.12812 (9)	1.07417 (6)	0.0180 (2)	
C31	0.55239 (12)	0.75514 (9)	0.87923 (6)	0.0191 (2)	
H31A	0.514984	0.737813	0.825853	0.023*	
H31B	0.460126	0.752590	0.902969	0.023*	
C32	0.60900 (13)	0.66285 (9)	0.89173 (6)	0.0234 (2)	
H32A	0.524286	0.586020	0.866974	0.035*	
H32B	0.633711	0.674000	0.944539	0.035*	
H32C	0.705539	0.668964	0.871761	0.035*	
C33	1.08165 (12)	0.74969 (9)	0.71392 (6)	0.0193 (2)	
H33A	1.047393	0.684652	0.734735	0.023*	
H33B	1.180674	0.811459	0.747120	0.023*	
C34	1.11428 (12)	0.70845 (9)	0.63836 (6)	0.0206 (2)	
H34A	1.158144	0.775297	0.619813	0.025*	
H34B	1.195547	0.674305	0.641710	0.025*	
C35	0.82914 (13)	0.65866 (9)	0.58539 (6)	0.0195 (2)	
H35A	0.730063	0.592981	0.555266	0.023*	
H35B	0.850732	0.721307	0.562431	0.023*	
C36	0.80354 (12)	0.70219 (9)	0.66243 (6)	0.0184 (2)	
H36A	0.717377	0.732118	0.660625	0.022*	
H36B	0.770777	0.637893	0.683949	0.022*	
O7	0.40275 (12)	0.71962 (9)	1.02567 (5)	0.0453 (3)	
O8	0.18454 (10)	0.59661 (7)	1.04995 (5)	0.0324 (2)	
N7	0.16228 (11)	0.77033 (8)	1.15200 (5)	0.01979 (18)	
N8	0.15170 (11)	0.85107 (8)	1.20891 (5)	0.02063 (18)	
C40	0.29378 (13)	0.79270 (9)	1.12391 (6)	0.0210 (2)	
C41	0.42495 (13)	0.89960 (10)	1.15102 (6)	0.0243 (2)	
H41	0.516498	0.914854	1.129385	0.029*	
C42	0.41628 (13)	0.98111 (9)	1.20973 (6)	0.0247 (2)	
H42	0.502470	1.054462	1.231155	0.030*	
C43	0.27586 (13)	0.95225 (9)	1.23692 (6)	0.0227 (2)	
H43	0.268519	1.008091	1.277777	0.027*	
C44	0.29505 (14)	0.69461 (10)	1.06044 (6)	0.0274 (2)	
O9	1.15796 (12)	0.79246 (7)	0.47867 (5)	0.0333 (2)	
O10	1.03207 (10)	0.59978 (7)	0.45318 (4)	0.02688 (18)	
N9	1.17088 (11)	0.55982 (8)	0.33985 (5)	0.01952 (18)	
N10	1.22934 (11)	0.53331 (8)	0.27920 (5)	0.02252 (19)	
C50	1.19622 (12)	0.66957 (9)	0.37419 (6)	0.0184 (2)	
C51	1.28665 (13)	0.76160 (9)	0.35105 (6)	0.0207 (2)	
H51	1.304587	0.839401	0.376648	0.025*	
C52	1.34837 (13)	0.73528 (10)	0.29006 (6)	0.0222 (2)	
H52	1.412129	0.794308	0.272192	0.027*	
C53	1.31435 (13)	0.61876 (10)	0.25507 (6)	0.0225 (2)	
H53	1.353835	0.599347	0.211819	0.027*	
C54	1.12238 (13)	0.69055 (9)	0.44173 (6)	0.0214 (2)	
H2	0.536 (2)	0.2757 (16)	0.4172 (10)	0.052 (5)*	
H3A	0.0276 (18)	0.3844 (13)	0.9055 (8)	0.032 (4)*	
H3B	0.144 (2)	0.4966 (15)	0.9675 (10)	0.047 (4)*	
H5A	0.966 (2)	1.2374 (15)	1.0694 (10)	0.053 (5)*	
H6A	0.9365 (17)	0.5543 (13)	0.5961 (8)	0.027 (3)*	
H6B	0.9885 (19)	0.6087 (14)	0.5361 (9)	0.043 (4)*	

### 4-(3-Carboxy-1-ethyl-6-fluoro-4-oxo-1,4-dihydroquinolin-7-yl)piperazin-1-ium pyridazine-3-carboxylate (2) Atomic displacement parameters (Å^2^)

**Table:**

	*U*^11^	*U*^22^	*U*^33^	*U*^12^	*U*^13^	*U*^23^
F1	0.0344 (3)	0.0128 (3)	0.0232 (3)	0.0077 (3)	0.0105 (3)	0.0033 (2)
O1	0.0236 (4)	0.0173 (3)	0.0216 (4)	0.0066 (3)	0.0102 (3)	0.0094 (3)
O2	0.0249 (4)	0.0210 (4)	0.0202 (4)	0.0080 (3)	0.0107 (3)	0.0088 (3)
O3	0.0261 (4)	0.0187 (4)	0.0194 (4)	0.0086 (3)	0.0077 (3)	0.0042 (3)
N1	0.0191 (4)	0.0135 (4)	0.0162 (4)	0.0051 (3)	0.0054 (3)	0.0055 (3)
N2	0.0187 (4)	0.0169 (4)	0.0160 (4)	0.0063 (3)	0.0060 (3)	0.0043 (3)
N3	0.0228 (5)	0.0192 (4)	0.0206 (5)	0.0017 (4)	0.0105 (4)	0.0013 (4)
C1	0.0178 (4)	0.0152 (5)	0.0159 (5)	0.0064 (4)	0.0036 (4)	0.0040 (4)
C2	0.0154 (4)	0.0172 (5)	0.0162 (5)	0.0057 (4)	0.0036 (4)	0.0053 (4)
C3	0.0143 (4)	0.0166 (5)	0.0183 (5)	0.0048 (4)	0.0030 (4)	0.0069 (4)
C4	0.0152 (4)	0.0157 (5)	0.0168 (5)	0.0046 (4)	0.0029 (4)	0.0051 (4)
C5	0.0183 (5)	0.0152 (5)	0.0188 (5)	0.0040 (4)	0.0045 (4)	0.0067 (4)
C6	0.0196 (5)	0.0119 (4)	0.0200 (5)	0.0050 (4)	0.0037 (4)	0.0023 (4)
C7	0.0150 (4)	0.0176 (5)	0.0159 (5)	0.0050 (4)	0.0035 (4)	0.0043 (4)
C8	0.0167 (4)	0.0159 (5)	0.0172 (5)	0.0042 (4)	0.0046 (4)	0.0062 (4)
C9	0.0150 (4)	0.0148 (5)	0.0165 (5)	0.0051 (4)	0.0028 (4)	0.0044 (4)
C10	0.0147 (4)	0.0187 (5)	0.0165 (5)	0.0046 (4)	0.0032 (4)	0.0053 (4)
C11	0.0258 (5)	0.0139 (5)	0.0186 (5)	0.0068 (4)	0.0089 (4)	0.0074 (4)
C12	0.0248 (5)	0.0192 (5)	0.0263 (6)	0.0032 (4)	0.0084 (4)	0.0098 (4)
C13	0.0192 (5)	0.0183 (5)	0.0220 (5)	0.0068 (4)	0.0060 (4)	0.0047 (4)
C14	0.0235 (5)	0.0173 (5)	0.0258 (6)	0.0055 (4)	0.0107 (4)	0.0027 (4)
C15	0.0244 (5)	0.0214 (5)	0.0175 (5)	0.0042 (4)	0.0064 (4)	0.0050 (4)
C16	0.0224 (5)	0.0178 (5)	0.0174 (5)	0.0064 (4)	0.0068 (4)	0.0054 (4)
F2	0.0194 (3)	0.0228 (3)	0.0206 (3)	0.0032 (2)	0.0096 (2)	0.0078 (2)
O4	0.0220 (4)	0.0165 (3)	0.0194 (4)	0.0007 (3)	0.0041 (3)	0.0024 (3)
O5	0.0262 (4)	0.0173 (4)	0.0194 (4)	0.0038 (3)	0.0053 (3)	0.0012 (3)
O6	0.0220 (4)	0.0228 (4)	0.0195 (4)	0.0078 (3)	0.0072 (3)	0.0042 (3)
N4	0.0152 (4)	0.0149 (4)	0.0160 (4)	0.0033 (3)	0.0041 (3)	0.0037 (3)
N5	0.0167 (4)	0.0173 (4)	0.0164 (4)	0.0065 (3)	0.0048 (3)	0.0042 (3)
N6	0.0237 (4)	0.0157 (4)	0.0204 (4)	0.0074 (4)	0.0094 (4)	0.0057 (3)
C21	0.0156 (4)	0.0190 (5)	0.0161 (5)	0.0063 (4)	0.0041 (4)	0.0058 (4)
C22	0.0175 (5)	0.0165 (5)	0.0147 (5)	0.0061 (4)	0.0028 (4)	0.0045 (4)
C23	0.0173 (5)	0.0160 (5)	0.0163 (5)	0.0048 (4)	0.0013 (4)	0.0062 (4)
C24	0.0171 (5)	0.0159 (5)	0.0159 (5)	0.0054 (4)	0.0030 (4)	0.0067 (4)
C25	0.0167 (5)	0.0151 (5)	0.0170 (5)	0.0025 (4)	0.0022 (4)	0.0067 (4)
C26	0.0159 (4)	0.0191 (5)	0.0183 (5)	0.0058 (4)	0.0065 (4)	0.0097 (4)
C27	0.0185 (5)	0.0168 (5)	0.0150 (5)	0.0074 (4)	0.0036 (4)	0.0064 (4)
C28	0.0172 (5)	0.0144 (4)	0.0174 (5)	0.0045 (4)	0.0031 (4)	0.0051 (4)
C29	0.0153 (4)	0.0161 (5)	0.0164 (5)	0.0055 (4)	0.0039 (4)	0.0069 (4)
C30	0.0192 (5)	0.0178 (5)	0.0168 (5)	0.0077 (4)	0.0021 (4)	0.0050 (4)
C31	0.0151 (4)	0.0162 (5)	0.0193 (5)	0.0003 (4)	0.0047 (4)	0.0021 (4)
C32	0.0223 (5)	0.0166 (5)	0.0257 (5)	0.0011 (4)	0.0050 (4)	0.0066 (4)
C33	0.0178 (5)	0.0204 (5)	0.0207 (5)	0.0080 (4)	0.0055 (4)	0.0067 (4)
C34	0.0195 (5)	0.0192 (5)	0.0237 (5)	0.0069 (4)	0.0092 (4)	0.0068 (4)
C35	0.0220 (5)	0.0179 (5)	0.0177 (5)	0.0076 (4)	0.0046 (4)	0.0041 (4)
C36	0.0173 (5)	0.0179 (5)	0.0181 (5)	0.0059 (4)	0.0045 (4)	0.0036 (4)
O7	0.0377 (5)	0.0459 (6)	0.0320 (5)	−0.0018 (4)	0.0214 (4)	−0.0013 (4)
O8	0.0347 (5)	0.0240 (4)	0.0257 (4)	0.0009 (4)	0.0143 (4)	−0.0015 (3)
N7	0.0217 (4)	0.0183 (4)	0.0162 (4)	0.0044 (3)	0.0035 (3)	0.0052 (3)
N8	0.0234 (4)	0.0189 (4)	0.0183 (4)	0.0078 (4)	0.0022 (3)	0.0051 (3)
C40	0.0217 (5)	0.0216 (5)	0.0156 (5)	0.0029 (4)	0.0035 (4)	0.0071 (4)
C41	0.0211 (5)	0.0253 (6)	0.0218 (5)	0.0015 (4)	0.0031 (4)	0.0108 (4)
C42	0.0246 (5)	0.0176 (5)	0.0243 (5)	0.0011 (4)	−0.0031 (4)	0.0072 (4)
C43	0.0270 (5)	0.0182 (5)	0.0205 (5)	0.0086 (4)	−0.0010 (4)	0.0046 (4)
C44	0.0255 (5)	0.0296 (6)	0.0181 (5)	0.0023 (5)	0.0076 (4)	0.0032 (4)
O9	0.0503 (5)	0.0188 (4)	0.0266 (4)	0.0105 (4)	0.0141 (4)	0.0018 (3)
O10	0.0360 (4)	0.0202 (4)	0.0244 (4)	0.0085 (3)	0.0155 (3)	0.0068 (3)
N9	0.0220 (4)	0.0180 (4)	0.0184 (4)	0.0069 (3)	0.0064 (3)	0.0060 (3)
N10	0.0263 (5)	0.0222 (5)	0.0216 (4)	0.0106 (4)	0.0094 (4)	0.0076 (4)
C50	0.0191 (5)	0.0171 (5)	0.0171 (5)	0.0056 (4)	0.0015 (4)	0.0051 (4)
C51	0.0219 (5)	0.0166 (5)	0.0208 (5)	0.0049 (4)	0.0010 (4)	0.0062 (4)
C52	0.0190 (5)	0.0234 (5)	0.0245 (5)	0.0047 (4)	0.0035 (4)	0.0132 (4)
C53	0.0221 (5)	0.0276 (6)	0.0211 (5)	0.0107 (4)	0.0077 (4)	0.0106 (4)
C54	0.0262 (5)	0.0190 (5)	0.0182 (5)	0.0082 (4)	0.0052 (4)	0.0053 (4)

### 4-(3-Carboxy-1-ethyl-6-fluoro-4-oxo-1,4-dihydroquinolin-7-yl)piperazin-1-ium pyridazine-3-carboxylate (2) Geometric parameters (Å, º)

**Table:**

F1—C6	1.3604 (11)	N6—C34	1.4878 (14)
O1—C3	1.2663 (12)	N6—H6A	0.882 (15)
O2—C10	1.3325 (12)	N6—H6B	0.993 (17)
O2—H2	0.946 (18)	C21—C22	1.3794 (14)
O3—C10	1.2151 (13)	C21—H21	0.9500
N1—C1	1.3377 (13)	C22—C23	1.4306 (14)
N1—C9	1.4002 (13)	C22—C30	1.4869 (14)
N1—C11	1.4826 (12)	C23—C24	1.4502 (14)
N2—C7	1.4055 (13)	C24—C25	1.4074 (14)
N2—C16	1.4713 (13)	C24—C29	1.4076 (14)
N2—C13	1.4760 (13)	C25—C26	1.3558 (15)
N3—C15	1.4815 (15)	C25—H25	0.9500
N3—C14	1.4866 (15)	C26—C27	1.4157 (14)
N3—H3A	0.921 (16)	C27—C28	1.3833 (14)
N3—H3B	0.987 (17)	C28—C29	1.4063 (14)
C1—C2	1.3769 (14)	C28—H28	0.9500
C1—H1	0.9500	C31—C32	1.5180 (15)
C2—C3	1.4329 (14)	C31—H31A	0.9900
C2—C10	1.4852 (14)	C31—H31B	0.9900
C3—C4	1.4519 (14)	C32—H32A	0.9800
C4—C9	1.4081 (14)	C32—H32B	0.9800
C4—C5	1.4099 (14)	C32—H32C	0.9800
C5—C6	1.3582 (14)	C33—C34	1.5141 (14)
C5—H5	0.9500	C33—H33A	0.9900
C6—C7	1.4167 (14)	C33—H33B	0.9900
C7—C8	1.3859 (14)	C34—H34A	0.9900
C8—C9	1.4040 (14)	C34—H34B	0.9900
C8—H8	0.9500	C35—C36	1.5193 (14)
C11—C12	1.5209 (15)	C35—H35A	0.9900
C11—H11A	0.9900	C35—H35B	0.9900
C11—H11B	0.9900	C36—H36A	0.9900
C12—H12A	0.9800	C36—H36B	0.9900
C12—H12B	0.9800	O7—C44	1.2380 (14)
C12—H12C	0.9800	O8—C44	1.2647 (14)
C13—C14	1.5152 (14)	N7—C40	1.3322 (14)
C13—H13A	0.9900	N7—N8	1.3471 (13)
C13—H13B	0.9900	N8—C43	1.3294 (14)
C14—H14A	0.9900	C40—C41	1.4021 (15)
C14—H14B	0.9900	C40—C44	1.5277 (16)
C15—C16	1.5211 (14)	C41—C42	1.3675 (17)
C15—H15A	0.9900	C41—H41	0.9500
C15—H15B	0.9900	C42—C43	1.3947 (17)
C16—H16A	0.9900	C42—H42	0.9500
C16—H16B	0.9900	C43—H43	0.9500
F2—C26	1.3581 (11)	O9—C54	1.2379 (13)
O4—C23	1.2659 (13)	O10—C54	1.2642 (13)
O5—C30	1.3299 (13)	N9—C50	1.3326 (13)
O5—H5A	0.970 (19)	N9—N10	1.3466 (12)
O6—C30	1.2159 (13)	N10—C53	1.3316 (14)
N4—C21	1.3377 (13)	C50—C51	1.3977 (14)
N4—C29	1.3956 (13)	C50—C54	1.5295 (14)
N4—C31	1.4830 (12)	C51—C52	1.3688 (16)
N5—C27	1.4036 (13)	C51—H51	0.9500
N5—C36	1.4672 (13)	C52—C53	1.3944 (16)
N5—C33	1.4793 (13)	C52—H52	0.9500
N6—C35	1.4828 (13)	C53—H53	0.9500
			
C10—O2—H2	105.0 (11)	C21—C22—C30	118.84 (9)
C1—N1—C9	120.23 (8)	C23—C22—C30	120.99 (9)
C1—N1—C11	119.03 (8)	O4—C23—C22	123.18 (9)
C9—N1—C11	120.71 (8)	O4—C23—C24	121.17 (9)
C7—N2—C16	116.04 (8)	C22—C23—C24	115.64 (9)
C7—N2—C13	115.01 (8)	C25—C24—C29	118.97 (9)
C16—N2—C13	110.47 (8)	C25—C24—C23	119.77 (9)
C15—N3—C14	112.12 (8)	C29—C24—C23	121.26 (9)
C15—N3—H3A	109.7 (9)	C26—C25—C24	119.57 (9)
C14—N3—H3A	109.6 (9)	C26—C25—H25	120.2
C15—N3—H3B	109.7 (10)	C24—C25—H25	120.2
C14—N3—H3B	105.5 (10)	C25—C26—F2	118.85 (9)
H3A—N3—H3B	110.2 (13)	C25—C26—C27	123.04 (9)
N1—C1—C2	123.24 (9)	F2—C26—C27	118.07 (9)
N1—C1—H1	118.4	C28—C27—N5	123.62 (9)
C2—C1—H1	118.4	C28—C27—C26	117.39 (9)
C1—C2—C3	120.57 (9)	N5—C27—C26	118.94 (9)
C1—C2—C10	118.39 (9)	C27—C28—C29	120.97 (9)
C3—C2—C10	121.03 (9)	C27—C28—H28	119.5
O1—C3—C2	122.70 (9)	C29—C28—H28	119.5
O1—C3—C4	121.68 (9)	N4—C29—C28	120.83 (9)
C2—C3—C4	115.62 (9)	N4—C29—C24	119.15 (9)
C9—C4—C5	118.69 (9)	C28—C29—C24	120.01 (9)
C9—C4—C3	120.98 (9)	O6—C30—O5	121.39 (9)
C5—C4—C3	120.29 (9)	O6—C30—C22	123.50 (9)
C6—C5—C4	119.57 (9)	O5—C30—C22	115.11 (9)
C6—C5—H5	120.2	N4—C31—C32	112.58 (8)
C4—C5—H5	120.2	N4—C31—H31A	109.1
C5—C6—F1	118.68 (9)	C32—C31—H31A	109.1
C5—C6—C7	123.23 (9)	N4—C31—H31B	109.1
F1—C6—C7	118.02 (9)	C32—C31—H31B	109.1
C8—C7—N2	123.12 (9)	H31A—C31—H31B	107.8
C8—C7—C6	116.95 (9)	C31—C32—H32A	109.5
N2—C7—C6	119.82 (9)	C31—C32—H32B	109.5
C7—C8—C9	121.23 (9)	H32A—C32—H32B	109.5
C7—C8—H8	119.4	C31—C32—H32C	109.5
C9—C8—H8	119.4	H32A—C32—H32C	109.5
N1—C9—C8	120.53 (9)	H32B—C32—H32C	109.5
N1—C9—C4	119.27 (9)	N5—C33—C34	109.10 (8)
C8—C9—C4	120.18 (9)	N5—C33—H33A	109.9
O3—C10—O2	121.33 (9)	C34—C33—H33A	109.9
O3—C10—C2	123.60 (9)	N5—C33—H33B	109.9
O2—C10—C2	115.05 (9)	C34—C33—H33B	109.9
N1—C11—C12	112.23 (8)	H33A—C33—H33B	108.3
N1—C11—H11A	109.2	N6—C34—C33	110.96 (8)
C12—C11—H11A	109.2	N6—C34—H34A	109.4
N1—C11—H11B	109.2	C33—C34—H34A	109.4
C12—C11—H11B	109.2	N6—C34—H34B	109.4
H11A—C11—H11B	107.9	C33—C34—H34B	109.4
C11—C12—H12A	109.5	H34A—C34—H34B	108.0
C11—C12—H12B	109.5	N6—C35—C36	111.66 (8)
H12A—C12—H12B	109.5	N6—C35—H35A	109.3
C11—C12—H12C	109.5	C36—C35—H35A	109.3
H12A—C12—H12C	109.5	N6—C35—H35B	109.3
H12B—C12—H12C	109.5	C36—C35—H35B	109.3
N2—C13—C14	110.37 (9)	H35A—C35—H35B	107.9
N2—C13—H13A	109.6	N5—C36—C35	108.86 (8)
C14—C13—H13A	109.6	N5—C36—H36A	109.9
N2—C13—H13B	109.6	C35—C36—H36A	109.9
C14—C13—H13B	109.6	N5—C36—H36B	109.9
H13A—C13—H13B	108.1	C35—C36—H36B	109.9
N3—C14—C13	110.92 (9)	H36A—C36—H36B	108.3
N3—C14—H14A	109.5	C40—N7—N8	120.36 (9)
C13—C14—H14A	109.5	C43—N8—N7	118.60 (9)
N3—C14—H14B	109.5	N7—C40—C41	122.35 (10)
C13—C14—H14B	109.5	N7—C40—C44	116.27 (9)
H14A—C14—H14B	108.0	C41—C40—C44	121.38 (10)
N3—C15—C16	110.98 (9)	C42—C41—C40	117.56 (10)
N3—C15—H15A	109.4	C42—C41—H41	121.2
C16—C15—H15A	109.4	C40—C41—H41	121.2
N3—C15—H15B	109.4	C41—C42—C43	117.34 (10)
C16—C15—H15B	109.4	C41—C42—H42	121.3
H15A—C15—H15B	108.0	C43—C42—H42	121.3
N2—C16—C15	109.52 (8)	N8—C43—C42	123.75 (10)
N2—C16—H16A	109.8	N8—C43—H43	118.1
C15—C16—H16A	109.8	C42—C43—H43	118.1
N2—C16—H16B	109.8	O7—C44—O8	127.75 (11)
C15—C16—H16B	109.8	O7—C44—C40	116.98 (10)
H16A—C16—H16B	108.2	O8—C44—C40	115.27 (10)
C30—O5—H5A	105.7 (11)	C50—N9—N10	120.27 (9)
C21—N4—C29	120.07 (8)	C53—N10—N9	118.77 (9)
C21—N4—C31	119.43 (8)	N9—C50—C51	122.53 (10)
C29—N4—C31	120.42 (8)	N9—C50—C54	116.33 (9)
C27—N5—C36	116.51 (8)	C51—C50—C54	121.14 (9)
C27—N5—C33	114.37 (8)	C52—C51—C50	117.41 (10)
C36—N5—C33	110.41 (8)	C52—C51—H51	121.3
C35—N6—C34	113.11 (8)	C50—C51—H51	121.3
C35—N6—H6A	109.5 (9)	C51—C52—C53	117.65 (10)
C34—N6—H6A	110.6 (9)	C51—C52—H52	121.2
C35—N6—H6B	107.0 (9)	C53—C52—H52	121.2
C34—N6—H6B	107.2 (10)	N10—C53—C52	123.33 (10)
H6A—N6—H6B	109.3 (13)	N10—C53—H53	118.3
N4—C21—C22	123.64 (9)	C52—C53—H53	118.3
N4—C21—H21	118.2	O9—C54—O10	127.80 (10)
C22—C21—H21	118.2	O9—C54—C50	117.26 (9)
C21—C22—C23	120.11 (9)	O10—C54—C50	114.93 (9)
			
C9—N1—C1—C2	−0.45 (15)	C29—C24—C25—C26	0.55 (14)
C11—N1—C1—C2	177.66 (9)	C23—C24—C25—C26	−178.39 (9)
N1—C1—C2—C3	−1.17 (15)	C24—C25—C26—F2	178.53 (8)
N1—C1—C2—C10	179.55 (9)	C24—C25—C26—C27	0.93 (15)
C1—C2—C3—O1	−179.84 (9)	C36—N5—C27—C28	16.86 (14)
C10—C2—C3—O1	−0.57 (15)	C33—N5—C27—C28	−113.97 (11)
C1—C2—C3—C4	0.37 (14)	C36—N5—C27—C26	−160.74 (9)
C10—C2—C3—C4	179.64 (9)	C33—N5—C27—C26	68.44 (12)
O1—C3—C4—C9	−177.82 (9)	C25—C26—C27—C28	−0.77 (15)
C2—C3—C4—C9	1.98 (14)	F2—C26—C27—C28	−178.38 (8)
O1—C3—C4—C5	0.19 (15)	C25—C26—C27—N5	176.98 (9)
C2—C3—C4—C5	179.98 (9)	F2—C26—C27—N5	−0.63 (14)
C9—C4—C5—C6	2.37 (15)	N5—C27—C28—C29	−178.52 (9)
C3—C4—C5—C6	−175.69 (9)	C26—C27—C28—C29	−0.89 (14)
C4—C5—C6—F1	177.88 (9)	C21—N4—C29—C28	−179.37 (9)
C4—C5—C6—C7	1.05 (16)	C31—N4—C29—C28	3.90 (14)
C16—N2—C7—C8	16.02 (14)	C21—N4—C29—C24	1.38 (14)
C13—N2—C7—C8	−115.08 (11)	C31—N4—C29—C24	−175.35 (9)
C16—N2—C7—C6	−160.06 (9)	C27—C28—C29—N4	−176.89 (9)
C13—N2—C7—C6	68.84 (12)	C27—C28—C29—C24	2.35 (15)
C5—C6—C7—C8	−3.70 (15)	C25—C24—C29—N4	177.10 (9)
F1—C6—C7—C8	179.45 (9)	C23—C24—C29—N4	−3.98 (14)
C5—C6—C7—N2	172.62 (9)	C25—C24—C29—C28	−2.15 (14)
F1—C6—C7—N2	−4.24 (14)	C23—C24—C29—C28	176.77 (9)
N2—C7—C8—C9	−173.25 (9)	C21—C22—C30—O6	0.10 (15)
C6—C7—C8—C9	2.93 (15)	C23—C22—C30—O6	−177.26 (9)
C1—N1—C9—C8	−175.75 (9)	C21—C22—C30—O5	179.27 (9)
C11—N1—C9—C8	6.16 (14)	C23—C22—C30—O5	1.91 (14)
C1—N1—C9—C4	2.81 (14)	C21—N4—C31—C32	−104.23 (11)
C11—N1—C9—C4	−175.27 (9)	C29—N4—C31—C32	72.52 (12)
C7—C8—C9—N1	178.89 (9)	C27—N5—C33—C34	−163.21 (8)
C7—C8—C9—C4	0.33 (15)	C36—N5—C33—C34	63.05 (10)
C5—C4—C9—N1	178.38 (9)	C35—N6—C34—C33	51.15 (11)
C3—C4—C9—N1	−3.58 (14)	N5—C33—C34—N6	−56.11 (11)
C5—C4—C9—C8	−3.05 (14)	C34—N6—C35—C36	−50.97 (11)
C3—C4—C9—C8	174.99 (9)	C27—N5—C36—C35	165.01 (8)
C1—C2—C10—O3	−5.51 (15)	C33—N5—C36—C35	−62.34 (10)
C3—C2—C10—O3	175.21 (9)	N6—C35—C36—N5	55.62 (11)
C1—C2—C10—O2	172.92 (9)	C40—N7—N8—C43	−1.04 (14)
C3—C2—C10—O2	−6.36 (14)	N8—N7—C40—C41	−0.61 (16)
C1—N1—C11—C12	−104.20 (11)	N8—N7—C40—C44	178.88 (9)
C9—N1—C11—C12	73.91 (12)	N7—C40—C41—C42	1.87 (16)
C7—N2—C13—C14	−166.31 (8)	C44—C40—C41—C42	−177.60 (10)
C16—N2—C13—C14	59.97 (11)	C40—C41—C42—C43	−1.44 (16)
C15—N3—C14—C13	53.04 (12)	N7—N8—C43—C42	1.44 (15)
N2—C13—C14—N3	−55.53 (11)	C41—C42—C43—N8	−0.16 (16)
C14—N3—C15—C16	−53.94 (11)	N7—C40—C44—O7	168.04 (11)
C7—N2—C16—C15	166.44 (8)	C41—C40—C44—O7	−12.46 (17)
C13—N2—C16—C15	−60.36 (11)	N7—C40—C44—O8	−12.10 (16)
N3—C15—C16—N2	57.09 (11)	C41—C40—C44—O8	167.41 (11)
C29—N4—C21—C22	1.70 (15)	C50—N9—N10—C53	0.93 (15)
C31—N4—C21—C22	178.46 (9)	N10—N9—C50—C51	−1.85 (15)
N4—C21—C22—C23	−2.14 (15)	N10—N9—C50—C54	178.92 (9)
N4—C21—C22—C30	−179.52 (9)	N9—C50—C51—C52	0.85 (16)
C21—C22—C23—O4	−179.58 (9)	C54—C50—C51—C52	−179.96 (9)
C30—C22—C23—O4	−2.26 (15)	C50—C51—C52—C53	0.96 (15)
C21—C22—C23—C24	−0.46 (14)	N9—N10—C53—C52	0.96 (16)
C30—C22—C23—C24	176.87 (8)	C51—C52—C53—N10	−1.90 (16)
O4—C23—C24—C25	1.50 (15)	N9—C50—C54—O9	172.86 (10)
C22—C23—C24—C25	−177.65 (9)	C51—C50—C54—O9	−6.38 (15)
O4—C23—C24—C29	−177.42 (9)	N9—C50—C54—O10	−6.61 (14)
C22—C23—C24—C29	3.44 (14)	C51—C50—C54—O10	174.15 (10)

### 4-(3-Carboxy-1-ethyl-6-fluoro-4-oxo-1,4-dihydroquinolin-7-yl)piperazin-1-ium pyridazine-3-carboxylate (2) Hydrogen-bond geometry (Å, º)

**Table:**

*D*—H···*A*	*D*—H	H···*A*	*D*···*A*	*D*—H···*A*
O2—H2···O1	0.946 (18)	1.610 (18)	2.5174 (10)	159 (2)
O5—H5*A*···O4	0.970 (19)	1.592 (18)	2.5208 (11)	159 (2)
N3—H3*B*···O8	0.987 (17)	1.685 (18)	2.6682 (12)	174 (2)
N6—H6*B*···O10	0.993 (17)	1.668 (17)	2.6591 (12)	176 (2)
N3—H3*A*···O8^i^	0.921 (16)	2.254 (15)	2.8557 (13)	122 (1)
N3—H3*A*···N7^i^	0.921 (16)	2.072 (15)	2.9552 (13)	160 (1)
N6—H6*A*···O10^ii^	0.882 (15)	2.112 (14)	2.7721 (12)	131 (1)
N6—H6*A*···N9^ii^	0.882 (15)	2.194 (15)	3.0060 (13)	153 (1)
C11—H11*B*···O9^iii^	0.99	2.46	3.2870 (13)	141
C11—H11*A*···F2^iii^	0.99	2.37	3.2083 (12)	142
C16—H16*B*···N8^i^	0.99	2.50	3.4430 (14)	158
C36—H36*B*···N10^ii^	0.99	2.49	3.4388 (14)	160
C41—H41···O6	0.95	2.45	3.1048 (13)	126
C51—H51···O3^iv^	0.95	2.31	3.1186 (13)	143

Symmetry codes: (i) −*x*, −*y*+1, −*z*+2; (ii) −*x*+2, −*y*+1, −*z*+1; (iii) *x*−1, *y*−1, *z*; (iv) *x*+1, *y*+1, *z*.

### 4-(3-Carboxy-1-ethyl-6-fluoro-4-oxo-1,4-dihydroquinolin-7-yl)piperazin-1-ium pyrimidine-5-carboxylate monohydrate (3) Crystal data

**Table:**

C_16_H_19_FN_3_O_3_^+^·C_5_H_3_N_2_O_2_^−^·H_2_O	*Z* = 2
*M**_r_* = 461.45	*F*(000) = 484
Triclinic, *P*1	*D*_x_ = 1.471 Mg m^−^^3^
*a* = 8.3657 (1) Å	Cu *K*α radiation, λ = 1.54184 Å
*b* = 9.5115 (1) Å	Cell parameters from 22879 reflections
*c* = 13.6068 (2) Å	θ = 3.3–76.5°
α = 76.490 (1)°	µ = 0.98 mm^−^^1^
β = 82.771 (1)°	*T* = 100 K
γ = 84.520 (1)°	Plate, colourless
*V* = 1041.92 (2) Å^3^	0.22 × 0.17 × 0.04 mm

### 4-(3-Carboxy-1-ethyl-6-fluoro-4-oxo-1,4-dihydroquinolin-7-yl)piperazin-1-ium pyrimidine-5-carboxylate monohydrate (3) Data collection

**Table:**

Rigaku, XtaLAB Synergy, Dualflex, HyPix diffractometer	4184 independent reflections
Radiation source: micro-focus sealed X-ray tube, PhotonJet (Cu) X-ray Source	3922 reflections with *I* > 2σ(*I*)
Mirror monochromator	*R*_int_ = 0.023
Detector resolution: 10.0000 pixels mm^-1^	θ_max_ = 77.0°, θ_min_ = 3.4°
ω scans	*h* = −10→10
Absorption correction: gaussian (CrysAlisPro; Rigaku OD, 2025)	*k* = −11→12
*T*_min_ = 0.660, *T*_max_ = 1.000	*l* = −17→16
31025 measured reflections	

### 4-(3-Carboxy-1-ethyl-6-fluoro-4-oxo-1,4-dihydroquinolin-7-yl)piperazin-1-ium pyrimidine-5-carboxylate monohydrate (3) Refinement

**Table:**

Refinement on *F*^2^	Primary atom site location: structure-invariant direct methods
Least-squares matrix: full	Hydrogen site location: mixed
*R*[*F*^2^ > 2σ(*F*^2^)] = 0.037	H atoms treated by a mixture of independent and constrained refinement
*wR*(*F*^2^) = 0.097	*w* = 1/[σ^2^(*F*_o_^2^) + (0.043*P*)^2^ + 0.5856*P*] where *P* = (*F*_o_^2^ + 2*F*_c_^2^)/3
*S* = 1.07	(Δ/σ)_max_ = 0.001
4184 reflections	Δρ_max_ = 0.31 e Å^−^^3^
319 parameters	Δρ_min_ = −0.22 e Å^−^^3^
0 restraints	

### 4-(3-Carboxy-1-ethyl-6-fluoro-4-oxo-1,4-dihydroquinolin-7-yl)piperazin-1-ium pyrimidine-5-carboxylate monohydrate (3) Special details

**Table:**

Geometry. All esds (except the esd in the dihedral angle between two l.s. planes) are estimated using the full covariance matrix. The cell esds are taken into account individually in the estimation of esds in distances, angles and torsion angles; correlations between esds in cell parameters are only used when they are defined by crystal symmetry. An approximate (isotropic) treatment of cell esds is used for estimating esds involving l.s. planes.

### 4-(3-Carboxy-1-ethyl-6-fluoro-4-oxo-1,4-dihydroquinolin-7-yl)piperazin-1-ium pyrimidine-5-carboxylate monohydrate (3) Fractional atomic coordinates and isotropic or equivalent isotropic displacement parameters (Å^2^)

**Table:**

	*x*	*y*	*z*	*U*_iso_*/*U*_eq_	
F1	1.00744 (10)	0.70135 (9)	0.46524 (6)	0.0275 (2)	
O1	0.65784 (11)	1.12025 (10)	0.28850 (7)	0.0207 (2)	
O2	0.49367 (12)	1.35975 (11)	0.25190 (7)	0.0245 (2)	
O3	0.43857 (14)	1.48539 (11)	0.37189 (8)	0.0320 (3)	
N1	0.61538 (13)	1.14104 (11)	0.58840 (8)	0.0167 (2)	
N2	0.96501 (13)	0.69701 (11)	0.67052 (8)	0.0171 (2)	
N3	1.06829 (14)	0.46490 (12)	0.83098 (9)	0.0204 (2)	
C1	0.55431 (15)	1.24892 (13)	0.51833 (10)	0.0183 (3)	
H1	0.498024	1.329758	0.540074	0.022*	
C2	0.56856 (15)	1.24917 (13)	0.41660 (10)	0.0176 (3)	
C3	0.65135 (15)	1.12979 (13)	0.38021 (9)	0.0164 (2)	
C4	0.72927 (14)	1.01952 (13)	0.45551 (9)	0.0154 (2)	
C5	0.82828 (15)	0.90495 (13)	0.42572 (10)	0.0184 (3)	
H5	0.843295	0.898475	0.356497	0.022*	
C6	0.90209 (15)	0.80378 (13)	0.49700 (10)	0.0186 (3)	
C7	0.88033 (15)	0.80219 (13)	0.60212 (9)	0.0161 (2)	
C8	0.78058 (15)	0.91432 (13)	0.63112 (9)	0.0160 (2)	
H8	0.760466	0.916324	0.701082	0.019*	
C9	0.70881 (14)	1.02496 (13)	0.55868 (9)	0.0152 (2)	
C10	0.49419 (16)	1.37499 (14)	0.34660 (10)	0.0214 (3)	
C11	0.58814 (16)	1.15048 (14)	0.69656 (10)	0.0195 (3)	
H11A	0.562787	1.054175	0.739384	0.023*	
H11B	0.493945	1.219228	0.705946	0.023*	
C12	0.73378 (17)	1.20006 (15)	0.73108 (10)	0.0231 (3)	
H12A	0.711638	1.203265	0.803005	0.035*	
H12B	0.756881	1.296925	0.690519	0.035*	
H12C	0.827264	1.132123	0.722081	0.035*	
C13	0.94337 (16)	0.54377 (13)	0.67222 (10)	0.0202 (3)	
H13A	0.947010	0.531123	0.601772	0.024*	
H13B	0.836598	0.516786	0.708405	0.024*	
C14	1.07576 (16)	0.44603 (14)	0.72533 (10)	0.0223 (3)	
H14A	1.061348	0.343682	0.726032	0.027*	
H14B	1.182542	0.471168	0.688327	0.027*	
C15	1.08134 (16)	0.61905 (14)	0.83281 (10)	0.0217 (3)	
H15A	1.190013	0.648806	0.802018	0.026*	
H15B	1.067965	0.629505	0.904143	0.026*	
C16	0.95380 (16)	0.71720 (14)	0.77477 (10)	0.0194 (3)	
H16A	0.845114	0.694564	0.809733	0.023*	
H16B	0.969299	0.819575	0.773344	0.023*	
O4	0.85628 (11)	0.18288 (11)	1.00834 (8)	0.0268 (2)	
O5	0.78038 (12)	0.40830 (11)	0.92836 (8)	0.0273 (2)	
N4	0.36819 (14)	0.09850 (13)	1.08628 (9)	0.0240 (3)	
N5	0.30900 (13)	0.29866 (13)	0.95040 (9)	0.0229 (2)	
C20	0.57755 (15)	0.24858 (14)	0.99701 (9)	0.0182 (3)	
C21	0.52305 (16)	0.13136 (15)	1.07032 (10)	0.0209 (3)	
H21	0.598467	0.071528	1.111149	0.025*	
C22	0.26994 (16)	0.18287 (15)	1.02331 (10)	0.0225 (3)	
H22	0.161053	0.157882	1.031252	0.027*	
C23	0.46306 (16)	0.33280 (14)	0.93893 (10)	0.0218 (3)	
H23	0.494853	0.416807	0.889780	0.026*	
C24	0.75307 (16)	0.28187 (14)	0.97704 (10)	0.0199 (3)	
O1W	0.92942 (15)	−0.08187 (14)	1.15420 (9)	0.0391 (3)	
H2	0.552 (3)	1.268 (3)	0.2514 (17)	0.060 (7)*	
H3A	0.965 (3)	0.432 (2)	0.8672 (16)	0.045 (5)*	
H3B	1.151 (2)	0.407 (2)	0.8647 (14)	0.036 (5)*	
H1W	0.887 (3)	0.007 (3)	1.1247 (17)	0.049 (6)*	
H2W	1.003 (3)	−0.115 (3)	1.105 (2)	0.077 (8)*	

### 4-(3-Carboxy-1-ethyl-6-fluoro-4-oxo-1,4-dihydroquinolin-7-yl)piperazin-1-ium pyrimidine-5-carboxylate monohydrate (3) Atomic displacement parameters (Å^2^)

**Table:**

	*U*^11^	*U*^22^	*U*^33^	*U*^12^	*U*^13^	*U*^23^
F1	0.0346 (5)	0.0214 (4)	0.0231 (4)	0.0141 (3)	−0.0008 (3)	−0.0059 (3)
O1	0.0254 (5)	0.0195 (5)	0.0166 (4)	0.0002 (4)	−0.0053 (4)	−0.0017 (3)
O2	0.0287 (5)	0.0205 (5)	0.0222 (5)	0.0021 (4)	−0.0094 (4)	0.0013 (4)
O3	0.0421 (6)	0.0182 (5)	0.0362 (6)	0.0111 (4)	−0.0183 (5)	−0.0050 (4)
N1	0.0187 (5)	0.0131 (5)	0.0180 (5)	0.0023 (4)	−0.0038 (4)	−0.0032 (4)
N2	0.0193 (5)	0.0117 (5)	0.0203 (5)	0.0027 (4)	−0.0062 (4)	−0.0027 (4)
N3	0.0166 (5)	0.0171 (5)	0.0246 (6)	0.0022 (4)	−0.0061 (4)	0.0017 (4)
C1	0.0180 (6)	0.0125 (6)	0.0243 (6)	0.0017 (5)	−0.0057 (5)	−0.0030 (5)
C2	0.0172 (6)	0.0137 (6)	0.0211 (6)	−0.0008 (5)	−0.0059 (5)	−0.0007 (5)
C3	0.0155 (6)	0.0141 (6)	0.0192 (6)	−0.0038 (5)	−0.0034 (5)	−0.0009 (5)
C4	0.0146 (6)	0.0134 (6)	0.0181 (6)	−0.0015 (5)	−0.0033 (5)	−0.0019 (5)
C5	0.0208 (6)	0.0165 (6)	0.0177 (6)	−0.0005 (5)	−0.0017 (5)	−0.0040 (5)
C6	0.0193 (6)	0.0135 (6)	0.0223 (6)	0.0037 (5)	−0.0002 (5)	−0.0055 (5)
C7	0.0165 (6)	0.0119 (6)	0.0193 (6)	−0.0014 (5)	−0.0040 (5)	−0.0014 (5)
C8	0.0178 (6)	0.0132 (6)	0.0170 (6)	0.0000 (5)	−0.0035 (5)	−0.0028 (5)
C9	0.0147 (6)	0.0116 (5)	0.0191 (6)	−0.0009 (5)	−0.0023 (5)	−0.0024 (4)
C10	0.0207 (6)	0.0163 (6)	0.0260 (7)	−0.0001 (5)	−0.0086 (5)	0.0002 (5)
C11	0.0219 (6)	0.0168 (6)	0.0188 (6)	0.0045 (5)	−0.0020 (5)	−0.0044 (5)
C12	0.0276 (7)	0.0190 (6)	0.0239 (7)	0.0009 (5)	−0.0062 (5)	−0.0063 (5)
C13	0.0251 (7)	0.0122 (6)	0.0237 (6)	0.0016 (5)	−0.0081 (5)	−0.0029 (5)
C14	0.0232 (7)	0.0157 (6)	0.0261 (7)	0.0046 (5)	−0.0051 (5)	−0.0019 (5)
C15	0.0215 (6)	0.0185 (6)	0.0247 (7)	−0.0012 (5)	−0.0089 (5)	−0.0007 (5)
C16	0.0228 (6)	0.0156 (6)	0.0197 (6)	0.0018 (5)	−0.0076 (5)	−0.0025 (5)
O4	0.0184 (5)	0.0263 (5)	0.0346 (5)	0.0034 (4)	−0.0064 (4)	−0.0045 (4)
O5	0.0220 (5)	0.0256 (5)	0.0310 (5)	−0.0045 (4)	−0.0044 (4)	0.0018 (4)
N4	0.0203 (6)	0.0260 (6)	0.0234 (6)	−0.0012 (5)	−0.0032 (4)	−0.0006 (5)
N5	0.0184 (5)	0.0243 (6)	0.0249 (6)	0.0022 (4)	−0.0062 (4)	−0.0027 (5)
C20	0.0185 (6)	0.0189 (6)	0.0176 (6)	0.0014 (5)	−0.0029 (5)	−0.0057 (5)
C21	0.0192 (6)	0.0226 (6)	0.0198 (6)	0.0018 (5)	−0.0050 (5)	−0.0022 (5)
C22	0.0177 (6)	0.0261 (7)	0.0233 (6)	−0.0003 (5)	−0.0028 (5)	−0.0050 (5)
C23	0.0202 (6)	0.0202 (6)	0.0235 (6)	0.0010 (5)	−0.0041 (5)	−0.0017 (5)
C24	0.0187 (6)	0.0223 (6)	0.0194 (6)	0.0003 (5)	−0.0042 (5)	−0.0053 (5)
O1W	0.0367 (6)	0.0383 (7)	0.0363 (6)	0.0112 (5)	−0.0060 (5)	−0.0009 (5)

### 4-(3-Carboxy-1-ethyl-6-fluoro-4-oxo-1,4-dihydroquinolin-7-yl)piperazin-1-ium pyrimidine-5-carboxylate monohydrate (3) Geometric parameters (Å, º)

**Table:**

F1—C6	1.3600 (14)	C11—H11A	0.9900
O1—C3	1.2663 (16)	C11—H11B	0.9900
O2—C10	1.3306 (17)	C12—H12A	0.9800
O2—H2	0.96 (2)	C12—H12B	0.9800
O3—C10	1.2136 (17)	C12—H12C	0.9800
N1—C1	1.3373 (16)	C13—C14	1.5179 (17)
N1—C9	1.3992 (16)	C13—H13A	0.9900
N1—C11	1.4830 (16)	C13—H13B	0.9900
N2—C7	1.4016 (15)	C14—H14A	0.9900
N2—C16	1.4656 (16)	C14—H14B	0.9900
N2—C13	1.4804 (15)	C15—C16	1.5202 (17)
N3—C14	1.4826 (18)	C15—H15A	0.9900
N3—C15	1.4868 (17)	C15—H15B	0.9900
N3—H3A	0.98 (2)	C16—H16A	0.9900
N3—H3B	0.94 (2)	C16—H16B	0.9900
C1—C2	1.3738 (18)	O4—C24	1.2503 (16)
C1—H1	0.9500	O5—C24	1.2566 (16)
C2—C3	1.4284 (18)	N4—C22	1.3355 (17)
C2—C10	1.4835 (17)	N4—C21	1.3409 (17)
C3—C4	1.4526 (17)	N5—C22	1.3344 (18)
C4—C9	1.4053 (17)	N5—C23	1.3404 (17)
C4—C5	1.4098 (18)	C20—C21	1.3817 (18)
C5—C6	1.3598 (18)	C20—C23	1.3899 (18)
C5—H5	0.9500	C20—C24	1.5093 (18)
C6—C7	1.4156 (18)	C21—H21	0.9500
C7—C8	1.3904 (17)	C22—H22	0.9500
C8—C9	1.4082 (17)	C23—H23	0.9500
C8—H8	0.9500	O1W—H1W	0.91 (2)
C11—C12	1.5115 (18)	O1W—H2W	0.94 (3)
			
C10—O2—H2	104.1 (14)	C11—C12—H12A	109.5
C1—N1—C9	119.98 (11)	C11—C12—H12B	109.5
C1—N1—C11	118.82 (10)	H12A—C12—H12B	109.5
C9—N1—C11	121.15 (10)	C11—C12—H12C	109.5
C7—N2—C16	116.27 (10)	H12A—C12—H12C	109.5
C7—N2—C13	116.59 (10)	H12B—C12—H12C	109.5
C16—N2—C13	109.80 (10)	N2—C13—C14	110.00 (10)
C14—N3—C15	111.23 (10)	N2—C13—H13A	109.7
C14—N3—H3A	108.0 (12)	C14—C13—H13A	109.7
C15—N3—H3A	109.6 (12)	N2—C13—H13B	109.7
C14—N3—H3B	110.5 (11)	C14—C13—H13B	109.7
C15—N3—H3B	109.7 (11)	H13A—C13—H13B	108.2
H3A—N3—H3B	107.7 (16)	N3—C14—C13	109.20 (11)
N1—C1—C2	123.55 (12)	N3—C14—H14A	109.8
N1—C1—H1	118.2	C13—C14—H14A	109.8
C2—C1—H1	118.2	N3—C14—H14B	109.8
C1—C2—C3	120.34 (11)	C13—C14—H14B	109.8
C1—C2—C10	118.33 (12)	H14A—C14—H14B	108.3
C3—C2—C10	121.32 (12)	N3—C15—C16	111.10 (10)
O1—C3—C2	122.66 (11)	N3—C15—H15A	109.4
O1—C3—C4	121.84 (11)	C16—C15—H15A	109.4
C2—C3—C4	115.51 (11)	N3—C15—H15B	109.4
C9—C4—C5	118.74 (11)	C16—C15—H15B	109.4
C9—C4—C3	121.10 (11)	H15A—C15—H15B	108.0
C5—C4—C3	120.16 (11)	N2—C16—C15	110.39 (11)
C6—C5—C4	119.37 (12)	N2—C16—H16A	109.6
C6—C5—H5	120.3	C15—C16—H16A	109.6
C4—C5—H5	120.3	N2—C16—H16B	109.6
C5—C6—F1	118.22 (11)	C15—C16—H16B	109.6
C5—C6—C7	123.74 (12)	H16A—C16—H16B	108.1
F1—C6—C7	117.99 (11)	C22—N4—C21	115.60 (11)
C8—C7—N2	123.02 (11)	C22—N5—C23	116.45 (11)
C8—C7—C6	116.51 (11)	C21—C20—C23	116.60 (12)
N2—C7—C6	120.34 (11)	C21—C20—C24	122.46 (11)
C7—C8—C9	121.21 (11)	C23—C20—C24	120.89 (12)
C7—C8—H8	119.4	N4—C21—C20	122.77 (12)
C9—C8—H8	119.4	N4—C21—H21	118.6
N1—C9—C4	119.12 (11)	C20—C21—H21	118.6
N1—C9—C8	120.58 (11)	N5—C22—N4	126.69 (12)
C4—C9—C8	120.30 (11)	N5—C22—H22	116.7
O3—C10—O2	121.35 (12)	N4—C22—H22	116.7
O3—C10—C2	123.52 (12)	N5—C23—C20	121.76 (12)
O2—C10—C2	115.12 (11)	N5—C23—H23	119.1
N1—C11—C12	112.01 (11)	C20—C23—H23	119.1
N1—C11—H11A	109.2	O4—C24—O5	126.42 (12)
C12—C11—H11A	109.2	O4—C24—C20	118.08 (12)
N1—C11—H11B	109.2	O5—C24—C20	115.49 (11)
C12—C11—H11B	109.2	H1W—O1W—H2W	108 (2)
H11A—C11—H11B	107.9		
			
C9—N1—C1—C2	4.53 (19)	C3—C4—C9—N1	−1.82 (17)
C11—N1—C1—C2	−178.12 (11)	C5—C4—C9—C8	−2.70 (18)
N1—C1—C2—C3	0.32 (19)	C3—C4—C9—C8	177.72 (11)
N1—C1—C2—C10	179.51 (11)	C7—C8—C9—N1	−176.73 (11)
C1—C2—C3—O1	174.88 (12)	C7—C8—C9—C4	3.75 (18)
C10—C2—C3—O1	−4.28 (19)	C1—C2—C10—O3	9.3 (2)
C1—C2—C3—C4	−5.52 (17)	C3—C2—C10—O3	−171.56 (13)
C10—C2—C3—C4	175.31 (11)	C1—C2—C10—O2	−171.44 (11)
O1—C3—C4—C9	−174.18 (11)	C3—C2—C10—O2	7.74 (18)
C2—C3—C4—C9	6.22 (17)	C1—N1—C11—C12	−100.30 (13)
O1—C3—C4—C5	6.24 (18)	C9—N1—C11—C12	77.01 (14)
C2—C3—C4—C5	−173.36 (11)	C7—N2—C13—C14	163.70 (11)
C9—C4—C5—C6	−0.51 (18)	C16—N2—C13—C14	−61.36 (13)
C3—C4—C5—C6	179.09 (11)	C15—N3—C14—C13	−56.66 (14)
C4—C5—C6—F1	−174.62 (11)	N2—C13—C14—N3	59.80 (14)
C4—C5—C6—C7	2.9 (2)	C14—N3—C15—C16	54.97 (14)
C16—N2—C7—C8	−4.76 (17)	C7—N2—C16—C15	−166.17 (10)
C13—N2—C7—C8	127.26 (13)	C13—N2—C16—C15	58.74 (13)
C16—N2—C7—C6	170.95 (11)	N3—C15—C16—N2	−55.66 (14)
C13—N2—C7—C6	−57.02 (16)	C22—N4—C21—C20	3.0 (2)
C5—C6—C7—C8	−1.85 (19)	C23—C20—C21—N4	−0.2 (2)
F1—C6—C7—C8	175.64 (11)	C24—C20—C21—N4	−177.82 (12)
C5—C6—C7—N2	−177.83 (12)	C23—N5—C22—N4	0.5 (2)
F1—C6—C7—N2	−0.35 (18)	C21—N4—C22—N5	−3.3 (2)
N2—C7—C8—C9	174.38 (11)	C22—N5—C23—C20	2.6 (2)
C6—C7—C8—C9	−1.49 (18)	C21—C20—C23—N5	−2.8 (2)
C1—N1—C9—C4	−3.67 (17)	C24—C20—C23—N5	174.92 (12)
C11—N1—C9—C4	179.05 (11)	C21—C20—C24—O4	18.43 (19)
C1—N1—C9—C8	176.80 (11)	C23—C20—C24—O4	−159.12 (13)
C11—N1—C9—C8	−0.48 (17)	C21—C20—C24—O5	−162.59 (13)
C5—C4—C9—N1	177.77 (11)	C23—C20—C24—O5	19.87 (18)

### 4-(3-Carboxy-1-ethyl-6-fluoro-4-oxo-1,4-dihydroquinolin-7-yl)piperazin-1-ium pyrimidine-5-carboxylate monohydrate (3) Hydrogen-bond geometry (Å, º)

**Table:**

*D*—H···*A*	*D*—H	H···*A*	*D*···*A*	*D*—H···*A*
O2—H2···O1	0.96 (2)	1.60 (3)	2.5234 (13)	160 (2)
N3—H3*A*···O5	0.98 (2)	1.67 (2)	2.6378 (15)	170 (2)
N3—H3*B*···N5^i^	0.94 (2)	1.93 (2)	2.8654 (16)	172 (2)
C1—H1···O3^ii^	0.95	2.48	3.2305 (16)	136
C11—H11*A*···N4^iii^	0.99	2.58	3.3630 (17)	136
C13—H13*A*···F1	0.99	2.20	2.8752 (15)	124
O1*W*—H1*W*···O4	0.91 (2)	2.04 (2)	2.8863 (16)	155 (2)
O1*W*—H2*W*···O4^iv^	0.94 (3)	2.01 (3)	2.9487 (17)	174 (2)

Symmetry codes: (i) *x*+1, *y*, *z*; (ii) −*x*+1, −*y*+3, −*z*+1; (iii) −*x*+1, −*y*+1, −*z*+2; (iv) −*x*+2, −*y*, −*z*+2.

## References

[bb1] Basavoju, S., Boström, D. & Velaga, S. P. (2006). *Cryst. Growth Des.* **6**, 2699–2708.

[bb2] Bernstein, J., Davis, R. E., Shimoni, L. & Chang, N. L. (1995). *Angew. Chem. Int. Ed. Engl.* **34**, 1555–1573.

[bb3] Bolla, G., Sarma, B. & Nangia, A. K. (2022). *Chem. Rev.* **122**, 11514–11603. 10.1021/acs.chemrev.1c00987 35642550

[bb5] Cremer, D. & Pople, J. A. (1975). *J. Am. Chem. Soc.* **97**, 1354–1358.

[bb4] da Paula Costa, G, Ferreira, P. O., Baptista, J. A., Pereira Silva, P. S., Castro, R. A. E., Caires, F. J. & Eusébio, M. E. S. (2026). *J. Solid State Chem.* **353**, 125698.

[bb6] de Paula Costa, G., Ferreira, P. O., Isquibola, G., dos Santos, J. G. & Caires, F. Jr (2025). *J. Therm. Anal. Calorim.* **150**, 14065–14076.

[bb7] Desiraju, G. R. (1989). In *Crystal Engineering. The Design of Organic Solids*. Amsterdam: Elsevier.

[bb8] Desiraju, G. R. (1995). *Angew. Chem. Int. Ed. Engl.* **34**, 2311–2327.

[bb9] Etter, M. C., MacDonald, J. C. & Bernstein, J. (1990). *Acta Cryst.* B**46**, 256–262. 10.1107/s0108768189012929 2344397

[bb10] Ferreira, P. O., de Almeida, A. C., dos Santos, C., Droppa, R., Junior, , Ferreira, F. F., Kogawa, A. C. & Caires, F. J. (2020). *Thermochim. Acta* **694**, 178782.

[bb11] Groom, C. R., Bruno, I. J., Lightfoot, M. P. & Ward, S. C. (2016). *Acta Cryst.* B**72**, 171–179. 10.1107/S2052520616003954 PMC4822653 27048719

[bb12] Gunnam, A. & Nangia, A. K. (2023). *Cryst. Growth Des.* **23**, 4198–4213.

[bb13] Holmes, B., Brogden, R. N. & Richards, D. M. (1985). *Drugs* **30**, 482–513. 10.2165/00003495-198530060-00003 3908074

[bb14] Huang, X.-F., Zhang, Z.-H., Zhang, Q.-Q., Wang, L.-Z., He, M.-Y., Chen, Q., Song, G.-Q., Wei, L., Wang, F. & Du, M. (2013). *Cryst­EngComm* **15**, 6090–6100.

[bb15] Lee, C. T. & Wong, E. C. (1988). *Scand. J. Infect. Dis. Suppl.* **56**, 49–54. 3150616

[bb16] Li, X.-A. & Jiang, C.-J. (2025). *Z. Kristallogr. New Cryst. Struct.* **240**, 509–510.

[bb17] Liang, D., Li, F., Duan, J., Sun, W. & Yu, X. (2024). *Antibiotics* **13**, 888. 10.3390/antibiotics13090888 PMC11429011 39335061

[bb18] Macrae, C. F., Sovago, I., Cottrell, S. J., Galek, P. T. A., McCabe, P., Pidcock, E., Platings, M., Shields, G. P., Stevens, J. S., Towler, M. & Wood, P. A. (2020). *J. Appl. Cryst.* **53**, 226–235. 10.1107/S1600576719014092 PMC6998782 32047413

[bb19] Reddy, J. S., Ganesh, S. V., Nagalapalli, R., Dandela, R., Solomon, K. A., Kumar, K. A., Goud, N. R. & Nangia, A. (2011). *J. Pharm. Sci.* **100**, 3160–3176. 10.1002/jps.22537 21394722

[bb20] Rigaku OD (2025). *CrysAlis PRO*. Rigaku Corporation, Yarnton, England.

[bb21] Sheldrick, G. M. (2015*a*). *Acta Cryst.* A**71**, 3–8.

[bb22] Sheldrick, G. M. (2015*b*). *Acta Cryst.* C**71**, 3–8.

[bb23] Spackman, M. A. & Jayatilaka, D. (2009). *CrystEngComm* **11**, 19–32.

[bb24] Spackman, M. A. & McKinnon, J. J. (2002). *CrystEngComm* **4**, 378–392.

[bb25] Spackman, P. R., Turner, M. J., McKinnon, J. J., Wolff, S. K., Grimwood, D. J., Jayatilaka, D. & Spackman, M. A. (2021). *J. Appl. Cryst.* **54**, 1006–1011. 10.1107/S1600576721002910 PMC8202033 34188619

[bb26] Spek, A. L. (2020). *Acta Cryst.* E**76**, 1–11. 10.1107/S2056989019016244 PMC6944088 31921444

[bb27] Sun, Y.-Y., Gu, Z.-N. & Jiang, C.-J. (2024). *Z. Kristallogr. New Cryst. Struct.* **239**, 865–867.

[bb28] Tabeta, H., Sawada, M., Honda, A., Takizawa, H. & Kikuchi, N. (1987). *Jpn J. Antibiot.* **40**, 1969–1974. 3448254

[bb29] Wang, S.-H., Yang, C.-Y., He, J.-S. & Jiang, C.-J. (2025). *Z. Kristallogr. New Cryst. Struct.* **240**, 895–896.

[bb30] Westrip, S. P. (2010). *J. Appl. Cryst.* **43**, 920–925.

[bb31] Xu, Y., Jiang, L. & Mei, X. (2014). *Acta Cryst.* B**70**, 750–760. 10.1107/S2052520614011718 25080254

[bb32] Yan, S.-W., Chen, H.-Y., Zhang, G.-J., Liao, Q. & Liang, Y.-C. (2011). *Acta Cryst.* E**67**, o909. 10.1107/S1600536811009524 PMC3099834 21754183

[bb33] Zeng, X., Zheng, C., Qiu, S., Jiang, X., Wang, X., Qian, K., Pan, R., Yang, J. & Ma, Y. (2024). *Cryst. Growth Des.* **24**, 4416–4427.

